# Prediction models of intravenous glucocorticoids therapy response in thyroid eye disease

**DOI:** 10.1530/ETJ-24-0122

**Published:** 2024-08-26

**Authors:** Haiyang Zhang, Shuo Wu, Shuyu Hu, Xianqun Fan, Xuefei Song, Tienan Feng, Huifang Zhou

**Affiliations:** 1 Department of Ophthalmology, Shanghai Ninth People’s Hospital, Shanghai Jiao Tong University School of Medicine, Shanghai, China; 2 Shanghai Key Laboratory of Orbital Diseases and Ocular Oncology, Shanghai, China; 3 Clinical Research Institute, Shanghai Jiao Tong University School of Medicine, Shanghai, China

**Keywords:** intravenous glucocorticoid therapy, meta-analysis, prediction models, systematic review, thyroid eye disease

## Abstract

**Background:**

Thyroid eye disease (TED) is an autoimmune orbital disease, with intravenous glucocorticoid (IVGC) therapy as the first-line treatment. Due to uncertain response rates and possible side effects, various prediction models have been developed to predict IVGC therapy outcomes.

**Methods:**

A thorough search was conducted in PubMed, Embase, and Web of Science databases. Data extraction included publication details, prediction model content, and performance. Statistical analysis was performed using R software, including heterogeneity evaluation, publication bias, subgroup analysis, and sensitivity analysis. Forest plots were utilized for result visualization.

**Results:**

Of the 12 eligible studies, 47 prediction models were extracted. All included studies exhibited a low-to-moderate risk of bias. The pooled area under the receiver operating characteristic curve (AUC) and the combined sensitivity and specificity for the models were 0.81, 0.75, and 0.79, respectively. In view of heterogeneity, multiple meta-regression and subgroup analysis were conducted, which showed that marker and modeling types may be the possible causes of heterogeneity (*P* < 0.001). Notably, imaging metrics alone (AUC = 0.81) or clinical characteristics combined with other markers (AUC = 0.87), incorporating with multivariate regression (AUC = 0.84) or radiomics analysis (AUC = 0.91), yielded robust and reliable prediction outcomes.

**Conclusion:**

This meta-analysis comprehensively reviews the predictive models for IVGC therapy response in TED. It underscores that integrating clinical characteristics with laboratory or imaging indicators and employing advanced techniques like multivariate regression or radiomics analysis significantly enhance the efficacy of prediction. Our research findings offer valuable insights that can guide future studies on prediction models for IVGC therapy in TED.

## Introduction

Thyroid eye disease (TED), also known as Graves’ ophthalmopathy, is the most common autoimmune orbital disease characterized by clinical presentations such as eyelid retraction, exophthalmos, periorbital edema, diplopia, and eye movement disorders (1). The disease can result in visual impairment and facial disfigurement, significantly impacting patients’ quality of life and mental health (2, 3). Based on the classical signs of inflammation – pain, redness, swelling, and impaired function – TED can be classified into active or inactive stages (4). Intravenous glucocorticoid (IVGC) therapy is the recommended first-line treatment for patients with active moderate-to-severe TED, as it effectively reduces inflammation (1). However, the response rate to IVGC therapy in clinical practice is only approximately 70–80% (5, 6). Related studies have indicated that a proportion of patients treated with IVGC therapy showed a poor treatment response and often experienced undesirable side effects, including hypertension, hyperglycemia, liver damage, and osteoporosis (5, 7, 8, 9). Therefore, it is crucial to assess patient response to IVGC therapy before initiating treatment for optimal results.

Numerous attempts have been made to construct prediction models for IVGC therapy response in TED, leveraging a comprehensive understanding of the disease’s pathogenesis (7). TED exhibits a range of complex disease traits, encompassing systemic immunoendocrine disturbances, genetic and environmental influences, as well as alterations in multiple orbital tissues (2, 10, 11). These clinical and biological indicators are closely intertwined with the likelihood of a favorable response to anti-inflammatory treatment, thus serving as valuable predictive factors for IVGC therapy outcomes. Therefore, various prediction models have been proposed for IVGC therapy response in TED, using clinical markers such as clinical activity score (CAS), smoking and disease duration, body fluid biomarkers such as thyroid-stimulating hormone receptor antibody, and imaging biomarkers like elevated signal intensity (SI) of orbital tissues (3, 6). However, there is no consensus on the specific markers used in the models and there are no standardized modeling methods, leading to conflicting conclusions on the effectiveness of markers. Additionally, there is a lack of systematic comparisons among different models, which hinders proper clinical management of TED and constrains future research endeavors in this field.

Given the current circumstances, the objective of this study was to conduct a comprehensive systematic review and meta-analysis to examine the effectiveness of existing prediction models in determining the response of patients with TED to IVGC therapy before initiation. By conducting an extensive search and applying strict selection criteria, we aimed to gather all accessible prediction models, providing a comprehensive overview of the current state of research in this field. Furthermore, subgroup analyses based on types of predictive markers, modeling methods, and IVGC protocol types were performed to gain deeper insights into the intriguing issues. Our goal was to identify key factors that demonstrate robust predictive capabilities and determine the overall effectiveness and specific modeling approaches, thereby establishing a framework for future advancements in reliable prediction models and facilitating prospective validation.

## Materials and methods

### Study design

This systematic review and meta-analysis were conducted following the statement of Preferred Reporting Items for a Systematic Review and Meta-analysis of Diagnostic Test Accuracy Studies (PRISMA-DTA) guidelines (12, 13). The study protocol was registered and approved on the international prospective register of systematic reviews, PROSPERO, before the start of the study (14).

### Literature search

A comprehensive search for relevant studies was performed in PubMed, Embase, and Web of Science databases until 6 May 2023. We built up the search strategy according to the PICO (population, intervention, control, and outcomes) principle. For our study, ‘P’ represented moderate-to-severe and active TED patients undergoing IVGC therapy, ‘I’ represented prediction methods as interventions, ‘C’ was unset, and ‘O’ represented the outcomes of prediction and diagnosis, such as the area under the curve (AUC), sensitivity, specificity, and accuracy. The following terms were used as Medical Subject Headings and natural language text words (including synonyms and closely related words) to identify relevant studies: ‘Glucocorticoids’ and ‘Graves Ophthalmopathy’ and ‘prediction’ (Supplementary Table 1, see the section on supplementary materials given at the end of this article). The references from the relevant reports or reviews were hand-searched for additional studies.

### Inclusion and exclusion criteria

The studies were included if they reported a prediction model relevant to IVGC therapy response for TED patients. The exclusion criteria were as follows: (a) the patients had other types of eye diseases; (b) the patients received other therapy combined with IVGC therapy; (c) article types were comments, abstracts, reviews, letters, and case reports; and (d) the studies did not report the AUC value or offer sufficient data to calculate the index. Endnote X9 was used to eliminate duplicates and facilitate screening. The full texts were evaluated if the title and abstract could not accurately identify the possibly eligible studies.

### Data extraction

All candidate articles were evaluated and extracted for eligibility by two independent authors (HYZ and SW). Disagreements were resolved by consensus after discussion with a third reviewer (HFZ). Data items extracted from each study included study characteristics, prediction model characteristics, specificity, sensitivity, and the receiver operating characteristic curve analysis results. To showcase the information in our research field comprehensively, if there were more than one model based on the same group of patients in the same study, all of them were incorporated into the analysis. A standardized form of extracted data is shown in Supplementary Table 2.

### Quality and bias assessments

The methodological quality assessment for all included studies was performed independently by two reviewers (HYZ and SYH) using the revised Quality Assessment Tool for Diagnostic Accuracy Studies (QUADAS)-2, and the discrepancy was resolved by consensus or a third reviewer (HFZ). The assessment included four domains: ‘Patient Selection’, ‘Index Test’, ‘Reference Standard’, and ‘Flow and Timing’. In each domain, several signal questions were answered as ‘Yes’, ‘No’, or ‘Unclear’ to assess the possibility of bias in this domain. According to the answers, the risk of bias of each domain and the application concern of the three domains were recorded and reported.

### Meta-analysis

Statistical analysis was carried out by utilizing the R (version 4.3.0) software. A summary receiver operating characteristic curve (SROC) was constructed for overall analysis, and the area under the SROC curve was calculated. We also conducted a meta-analysis of the models (38/47) with reported sensitivity and specificity and employed the forest plots for visualization.

### Assessment of heterogeneity

Cochran’s *Q* test and Higgins’s *I*-squared metric were undertaken to assess the heterogeneity of the included trials. Heterogeneity *P* < 0.05 indicates significant heterogeneity across studies. The *I*^2^ value of 0–25% represents low heterogeneity, 25–50% moderate heterogeneity, and >50% high heterogeneity. When heterogeneity was observed, the random-effects model was used. Otherwise, the fixed-effects model (common-effect model) was adopted. Besides, the multiple meta-regression was used to test whether there were certain confounders of the outcome and whether different model categories confound the effect size, influencing the heterogeneity.

### Assessment of publication bias

The possibility of publication bias was assessed by the Egger test (*P* < 0.05 was considered statistically significant) and visual inspection of the funnel plots. The trim-and-fill method was used to estimate potentially missing studies due to publication bias in the funnel plot, obtain adjusted random-effects pooled AUCs, and test our primary analysis on the publication bias.

### Subgroup analysis

Subgroup analysis was used to account for the impact of different model categories on heterogeneity. We conducted subgroup analyses based on the modeling, marker, and IVGC protocol types separately. A meta-analysis was conducted to estimate the effect of model performance. The forest plots were used to visualize the results, including statistics for the AUC and their standard error (s.e.). Subgroup analyses based on modeling types (univariate, multivariate regression, or radiomics analysis) and marker types (imaging metrics, clinical characteristics, clinical characteristics combined with laboratory indexes, or clinical features combined with imaging metrics) were separately conducted to investigate the origin of study heterogeneity. The analysis of intergroup differences was performed by R (version 4.3.0) software.

### Sensitivity analysis

To explore the stability of meta-analysis results, sensitivity analysis was performed by eliminating each study included one by one.

## Results

**Figure 1:**
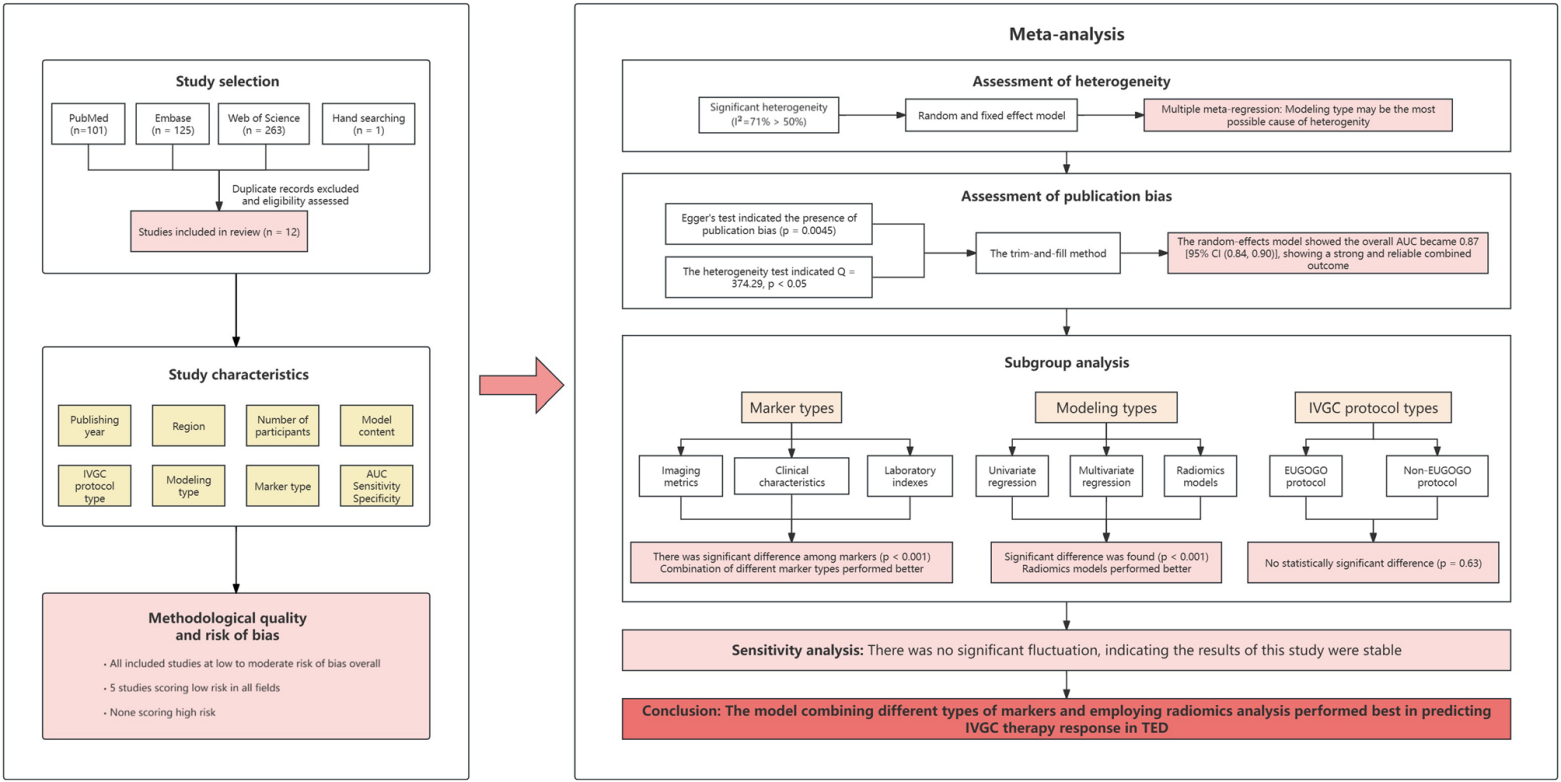
The research process and main results. AUC, the area under the curve; EUGOGO, European Group on Graves’ Orbitopathy (total 4.5 g methylprednisolone: 500 mg weekly for the first 6 weeks followed by 250 mg weekly for the next 6 weeks); IVGC, intravenous glucocorticoid.

The research process and main results are illustrated in Fig. 1.

### Study selection

**Figure 2:**
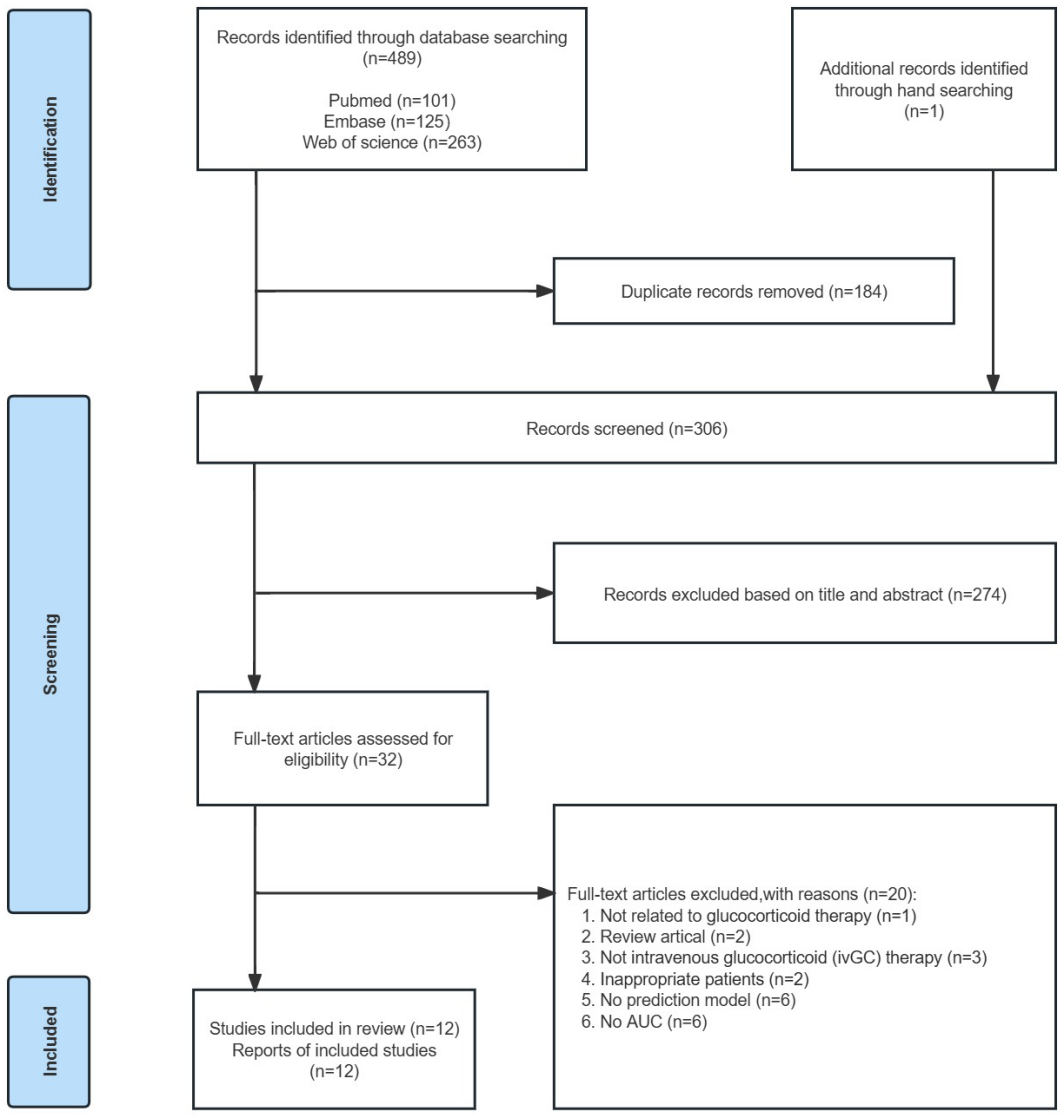
Flow diagram of screening stages. AUC, the area under the curve.

The search yielded 101 studies from PubMed, 125 from Embase, and 263 from Web of Science. After excluding 184 duplicates, 32 studies were selected for full-text review. Finally, the meta-analysis included 47 models from 12 articles with 801 patients. Reasons for exclusion at this stage were recorded and reported. The complete literature search and screen flowchart according to the PRISMA statement are summarized in Fig. 2. Reasons for exclusion at this stage were recorded and reported.

### Study characteristics

Characteristics of 12 eligible studies (6, 11, 15, 16, 17, 18, 19, 20, 21, 22, 23, 24) are presented in Supplementary Table 2. Eight out of 12 (66.7%) were published from 1992 to 2022. Eleven out of 12 (91.7%) originated from China. Forty-seven models from 12 studies were included, integrating predictive markers like clinical characteristics, laboratory indexes, and imaging metrics. The distribution of model types was as follows: 10/47 (20.8%) used clinical characteristics alone, 13/47 (27%) combined clinical characteristics with imaging metrics, 3/47 (6.2%) combined clinical features with laboratory indexes, and 21/47 (43.7%) relied solely on imaging metrics alone. AUC was the primary evaluation indicator, with 29/47 (61.7%) based on multivariate regression analysis, 14/47 (29.8%) on univariate regression analysis, and 4/47 (8.5%) on radiomics analysis. IVGC protocol types varied: 34/47 (72.3%) applied EUGOGO protocol, while only 13/47 (27.7%) applied non-EUGOGO protocol. Response definition varied, focusing on CAS score reduction and improvement in ocular symptoms like proptosis and visual acuity. Evaluation timing was predominantly 6 months after therapy (41.7%). Slight differences were observed in TED activity and severity definitions.

### Methodological quality and risk of bias

**Figure 3:**
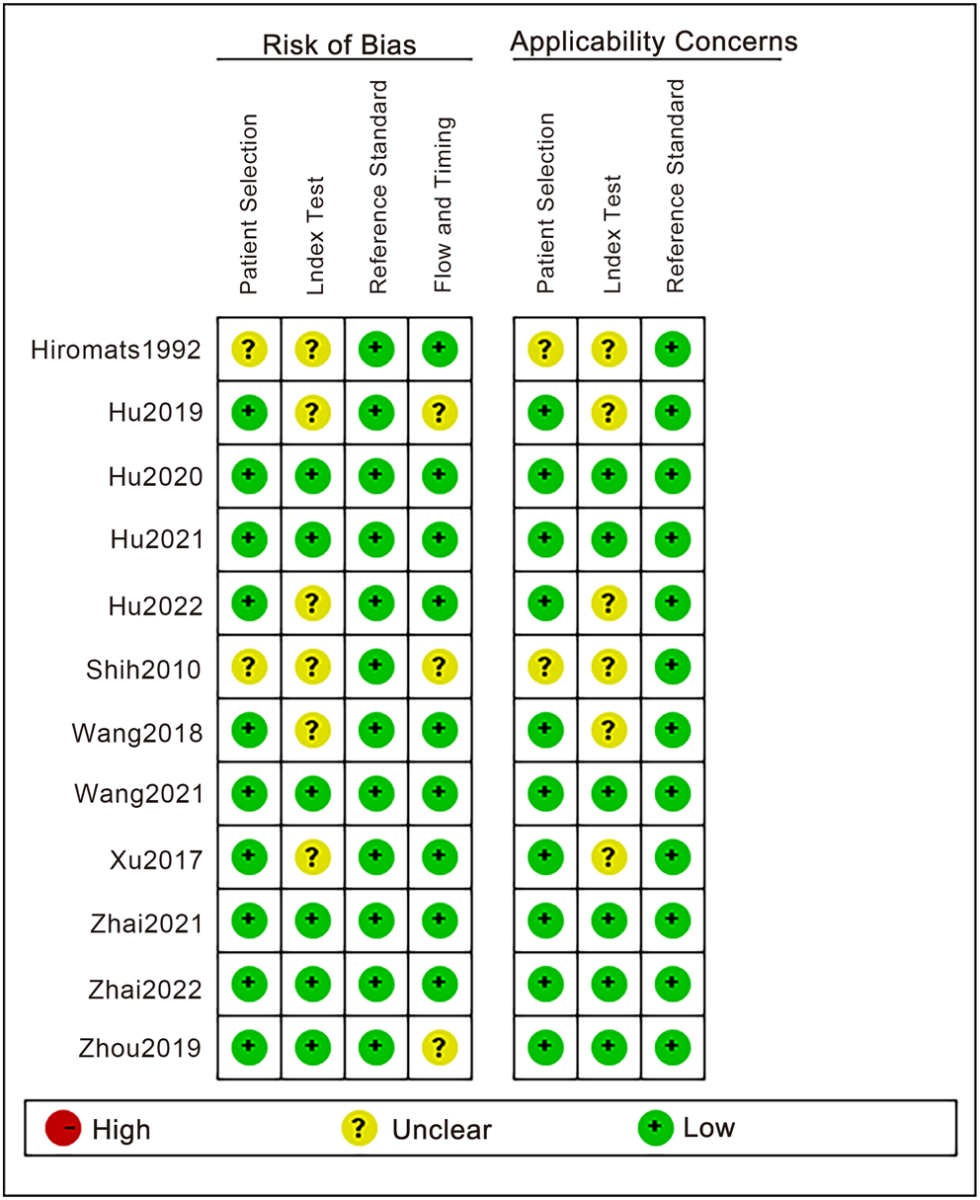
The methodological quality of the 12 included studies.

The methodological quality of the 12 included studies is shown in Fig. 3. According to the QUADAS-2 tool, all included studies were assessed as having an overall low-to-moderate risk of bias, with five studies rated as low risk in all domains and none rated as high risk. The primary factor contributing to the risk of bias is the absence of blinding during the study, as six studies failed to specify whether the interpretation of index test results was conducted without knowledge of the reference standard results. Furthermore, the absence of a specific time frame for referencing the standard after IVGC therapy and the lack of clear exclusions also contribute significantly to bias.

### Meta-analysis

**Figure 4:**
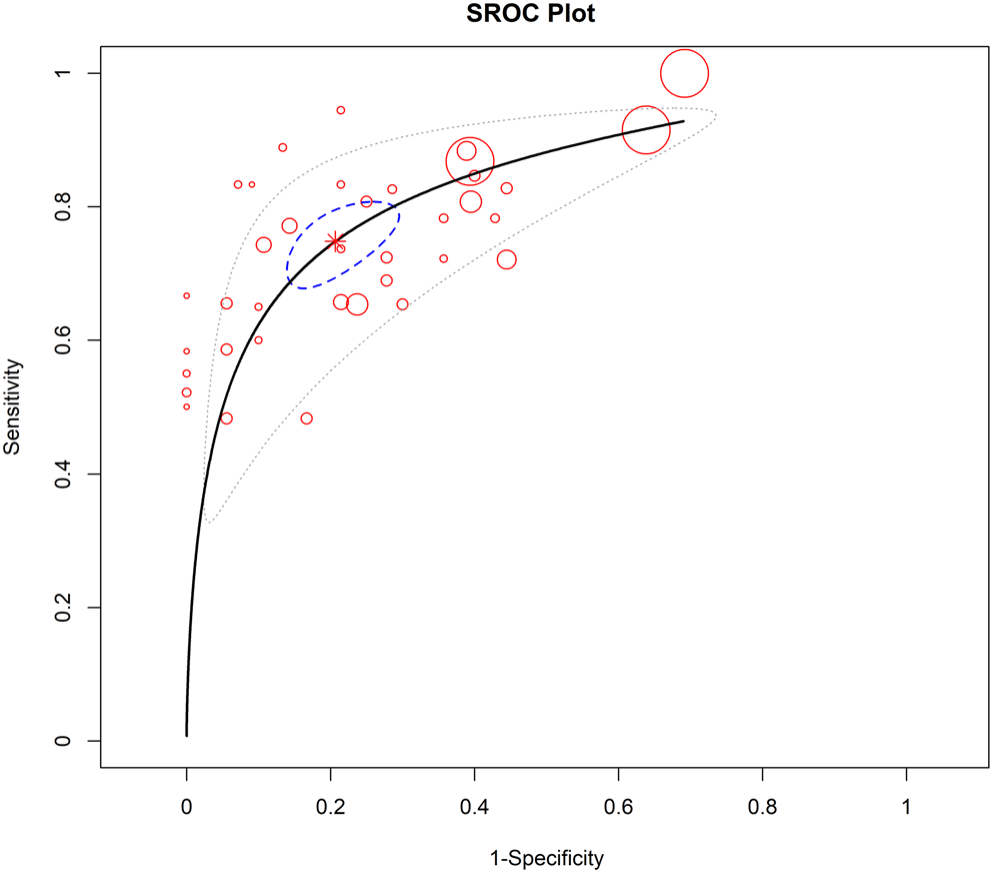
The SROC curve of prediction models of IVGC therapy response in TED. SROC, summary receiver operating characteristic curve; IVGC, intravenous glucocorticoid.

All 47 models demonstrated successful prediction capabilities, with the pooled AUC (Fig. 4 and Supplementary Figure 7) and the combined sensitivity (Supplementary Figure 8) and specificity (Supplementary Figure 9) of 0.81 (95% CI (0.78–0.83)), *I*^2^ = 71%, *P* < 0.01 for the Cochran’s *Q* test), 0.75 (95% CI (0.70, 0.79)), and 0.79 (95% CI (0.74, 0.84)), respectively.

### Assessment of heterogeneity

Higgins *I*^2^ test demonstrated significant heterogeneity for our primary outcome analysis (*I*^2^ = 71% > 50%). According to heterogeneity, a random-effects model was performed to analyze the differences within and between studies. Random- and fixed-effects model results are shown in Supplementary Figure 7. Multiple meta-regression for the different model types confirmed that modeling types may have been the most possible causes of heterogeneity (*P* < 0.001) (Table 1).

**Table 1:** Results of multiple meta-regression analysis.

	Coefficients	SE	*t*	*P*	*τ*^2^	*I*^2^-Residual	*R*^2^
					0.0038	45.39%	55.34%
Group 1 model type	0.1348	0.0263	5.124	<0.001			
Group 2 marker type	0.0145	0.0117	1.236	0.2165			
Group 3 IVGC protocol type	0.0492	0.0332	1.4825	0.1382			

IVGC, intravenous glucocorticoid; *I*^2^-Residual, unexplained heterogeneity as a percentage of total variation; *R*^2^, proportion of heterogeneity explained by the model; SE, standard error; *τ*^2^, the estimated amount of residual heterogeneity.

### Assessment of publication bias

**Figure 5:**
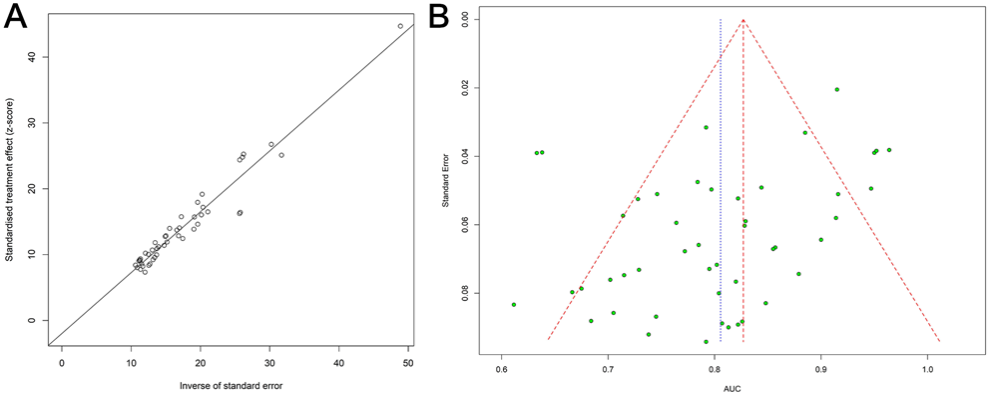
(A) Publication bias assessment by the Egger linear regression test. (B) Publication bias assessment funnel plot by the Egger linear regression test (*P* = 0.0045). AUC, the area under the curve.

**Figure 6:**
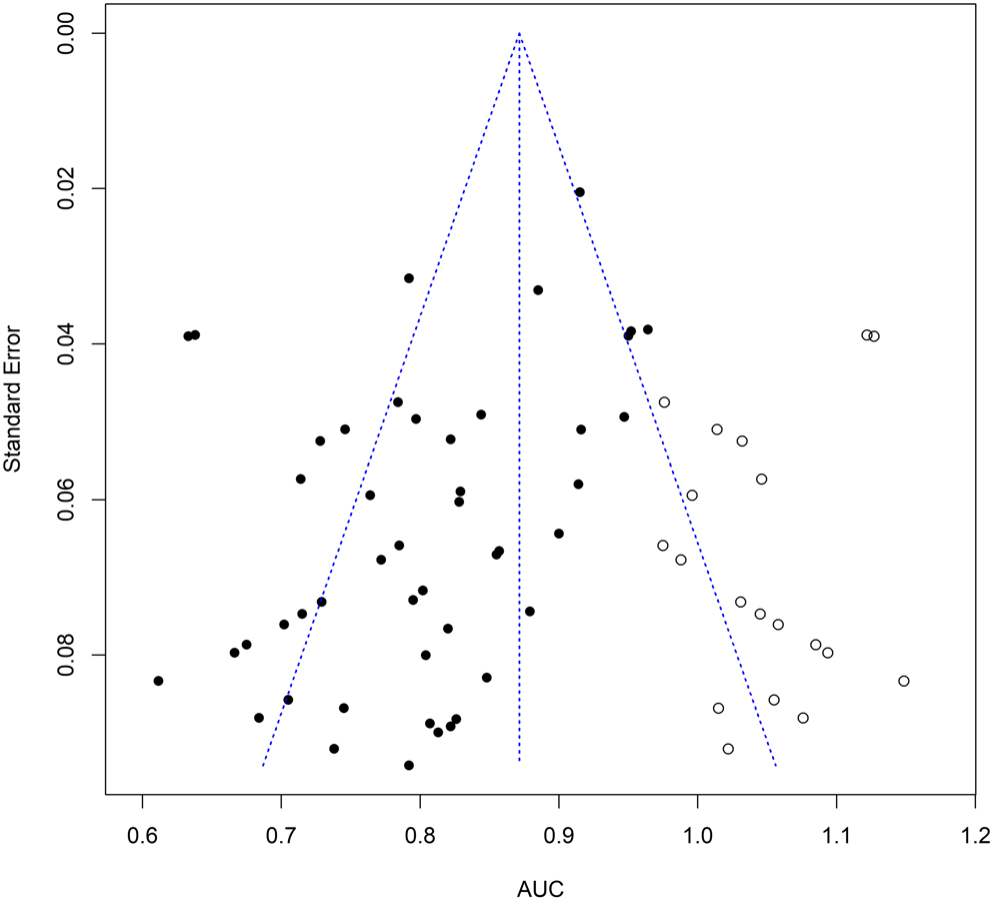
Funnel plot after applying a trim-and-fill method. AUC, the area under the curve.

Publication bias was detected by Egger’s test (*P* = 0.0045) and the visual inspection of the funnel plots (Fig. 5). The trim-and-fill method was used to test our primary analysis of the publication bias (Fig. 6). Nineteen missing studies were filled in the plot. The heterogeneity test revealed a result of *Q* = 374.29, *P* < 0.05. The random-effects model was employed, and the overall AUC became 0.87 (95% CI (0.84, 0.90)), indicating a strong and reliable combined outcome.

### Subgroup analysis

Subgroup analysis based on marker types, modeling types, and IVGC protocol types was separately conducted (Table 2). There was a significant difference among imaging metrics (*I*^2^ = 53%, AUC = 0.81, 95% CI (0.77–0.85)), clinical characteristics (*I*^2^ = 55%, AUC = 0.72, 95% CI (0.67–0.77)), clinical characteristics combined with laboratory indexes (*I*^2^ = 69%, AUC = 0.87, 95% CI (0.80–0.94)), and clinical characteristics combined with imaging metrics (*I*^2^ = 47%, AUC = 0.87, 95% CI (0.84–0.90)) (*P* < 0.001). However, the combined results of clinical characteristics and laboratory indices or imaging metrics were not statistically significant (*P* = 0.086). There was a significant difference among univariate (*I*^2^ = 39%, AUC = 0.72, 95% CI (0.69–0.75)) and multivariate regression (*I*^2^ = 51%, AUC = 0.84, 95% CI (0.81–0.87)) and radiomics models (*I*^2^ = 0%, AUC = 0.91, 95% CI (0.86–0.96)) (*P* < 0.001). However, the difference between EUGOGO protocol and non-EUGOGO protocol models was not statistically significant (*P* = 0.63).

**Table 2:** Subgroup meta-analysis of prediction models of IVGC therapy response in TED.

Subgroup/items	AUC	95% CI	*I*^2^	*P*
Group 1 modeling type			71%	<0.01
Univariate regression	0.72	0.69; 0.75	39%	
Multivariate regression	0.84	0.81; 0.87	51%	
Radiomics	0.91	0.86; 0.96	0%	
Group 2 marker type			71%	<0.01
Clinical characteristics	0.72	0.67; 0.77	56%	
Clinical characteristics + laboratory indexes	0.87	0.80; 0.94	69%	
Imaging metrics	0.81	0.77; 0.85	53%	
Clinical features + imaging metrics	0.87	0.84; 0.90	47%	
Group 3 IVGC protocol type				0.63
EUGOGO protocol	0.81	0.78; 0.84	54%	
Non-EUGOGO protocol	0.79	0.73; 0.86	86%	

AUC, the area under the curve; EUGOGO, European Group on Graves’ Orbitopathy (total 4.5 g methylprednisolone: 500 mg weekly for the first 6 weeks, followed by 250 mg weekly for the next 6 weeks); IVGC, intravenous glucocorticoid; *I*^2^, indicates heterogeneity.

### Sensitivity analysis

The results for the sensitivity analysis showed that the AUC fluctuation ranged from 0.80 to 0.81 after eliminating each study one by one. There was no significant difference compared with the total AUC, indicating that the results of this meta-analysis were stable and did not rely solely on the data of a particular study (Supplementary Figure 6).

## Discussion

The prediction of IVGC therapy response in TED has relied on the disease stage – active or inactive – mainly assessed by CAS, with disease duration sometimes considered. However, this is inherently subjective and lacks precision, thereby failing to fully meet the demands of effective treatment. The emergence of novel predictive markers and modeling methods offers promising new possibilities in this field. Studies have explored the predictive power of various factors, including clinical characteristics, imaging metrics, and laboratory indexes, for patients with TED undergoing IVGC therapy. Nonetheless, the lack of consensus on specific markers has led to varying conclusions on the model performance. Therefore, there is an urgent need for reliable prediction models for IVGC response in TED. In our study, we conducted a meta-analysis that revealed a satisfactory pooled diagnostic AUC of 0.81. However, the results of models based on different indicators varied significantly, warranting further exploration to develop simpler and more accurate prediction models for guiding clinical decision-making in TED patients undergoing IVGC therapy.

Our meta-analysis reveals notable heterogeneity among the encompassed studies. The variations in predictive markers and modeling techniques have a significant impact on the efficiency of prediction models (25) and contribute to observed heterogeneity. Subgroup analysis focusing on distinct marker types and modeling types has substantiated our conjecture.

A significant difference was observed based on marker types among clinical characteristics, imaging metrics, and laboratory indexes. The model using only clinical characteristics performed poorly, indicating insufficiency in capturing IVGC response. Taking CAS as an example, first, the subjectivity of the CAS leads to differences between scorers (26). Secondly, the CAS primarily reflects the condition of the periocular regions and may not fully represent the condition of the posterior lesions (27). Additionally, the binary results and recall bias of CAS scoring reduce the accuracy of the information (27). Therefore, it is imperative to identify more useful and reliable indicators, such as using MRI metrics, to complement CAS and enhance precision in evaluating inflammation and predicting IVGC therapy response.

Combining clinical characteristics with laboratory indexes or imaging metrics yielded better results than models using only clinical features, enhancing predictive performance. This approach incorporates diverse indicators from various categories for comprehensive patient assessment, leading to more reliable conclusions. Both the laboratory indexes (28) and imaging metrics (29) are crucial for accurately predicting the response of TED patients to IVGC therapy, closely related to the autoimmune nature and orbital tissue changes of TED (30).

TED, an autoimmune disease, is characterized by immunologic and inflammatory biomarker alterations in the blood (17, 31). Some of these biomarkers have proven effective in predicting responses to anti-inflammatory treatments. For instance, Mysliwiec *et al*. (32). observed that increased concentrations of circulating CXCL9 reflect the activity of orbital inflammation and serve as predictive markers for IVGC treatment response. Nonetheless, laboratory indexes currently suffer from limited specificity and inconsistent detection levels in clinical applications. Future research should explore more dependable indicators and conduct multicenter studies on laboratory indexes to predict treatment response and enhance marker applicability.

In recent years, researchers have discovered that MRI offers an intuitive visualization of the disease-affected region and pathological changes, playing a pivotal role in the diagnosis and treatment of TED (33). For example, Hu *et al*. found structural MRI-based quantitative measurements at extraocular muscle, orbital fat, and lacrimal gland, especially the minimum SI ratio of extraocular muscle and lacrimal gland herniation/orbital fat thickness ratio, together with disease duration, performed well to predict the response of IVGC in active and moderate-severe patients (17). However, MRI usage is constrained by its high cost and uneven availability across various regions. Consequently, MRI research in TED is mainly conducted in certain Asian countries, such as China and Japan, where MRI is highly prioritized in TED management. Considering its indispensable role in the management of TED, we advocate for the broader application of MRI. Ongoing efforts are being made to overcome these challenges, including innovations focused on creating more affordable and portable MRI systems, such as low-field MRI with improved image quality based on artificial intelligence technology (34). With increasing advancements in MRI technology, it is anticipated that its prevalence in TED management will rise in the future, facilitating precise IVGC therapy response prediction.

Furthermore, a significant difference based on modeling types was observed. Notably, the univariate regression was less powerful than the multivariate regression models. This highlights the complexity of TED and emphasizes the need to consider multiple factors to draw accurate and robust conclusions. Radiomics analysis exhibited the most robust predictive performance compared to the other types. Radiomics in predicting therapeutic response for TED patients to IVGC therapy shows promising results (35). It enables noninvasive tissue heterogeneity profiling through high-dimensional quantitative data extracted from radiological images (27, 36). Furthermore, the application of advanced modeling techniques like machine learning and artificial intelligence (37) empowers the construction of the models. For instance, an inquiry into the determinants influencing the prediction model of responsivity to IVGC treatment in TED patients was conducted employing the SHapley Additive exPlanation methodology (38, 39). It is worth mentioning that the best-performing models selected from each study are all constructed based on multivariate regression or radiomics analysis as well. Consequently, integrating these methodologies with high-throughput approaches like radiomics analysis presents a viable avenue for enhancing both the predictive capability and interpretability of the models.

Other factors, such as evaluation timelines and treatment protocols, may also contribute to observed heterogeneity, necessitating further investigation in the future. Achieving uniformity in this field is challenging due to the lack of consensus on evaluation criteria and timing in TED treatments. This issue reflects a broader challenge within the industry (3).

Moreover, in addition to objective clinical indicators, patient-reported outcomes should also be an essential part of the effectiveness assessment (40). While improved proptosis or CAS may be cited as treatment success, if patients do not experience a meaningful boost in their quality of life, such improvements may not be perceived as truly successful (41). Currently, there is a lack of research in this area, and future studies need to be conducted to address this gap.

Several limitations should be acknowledged. First, the number of included studies and the sample size of individual studies were relatively small, which may limit the generalizability of the findings. Secondly, the quality of the examined literature could be improved, highlighting the need for future high-quality prospective cohort studies with larger sample sizes. Thirdly, despite our comprehensive search efforts, the included studies were confined to China and Japan. However, we believe that the insights gained from this research have a degree of general applicability for understanding IVGC therapy response prediction in TED. Future multicenter studies across diverse regions are anticipated to further validate and expand the reach of our findings to a wider patient demographic.

In summary, our meta-analysis provides an overview of IVGC therapy prediction models in TED. The study results indicate that there is a significant variance in diagnostic accuracy among the various models currently in use. Combining clinical characteristics with laboratory indexes or imaging metrics and employing modeling techniques like multivariate regression or radiomics analysis contribute significantly to enhancing the efficacy of prediction models. Looking ahead, the identification of more sensitive biomarkers, the integration of advanced modeling methods like machine learning and artificial intelligence with high-throughput approaches, and the establishment of consensus on evaluating TED treatment are crucial directions for enhancing model effectiveness in the future.

## Supplementary Materials

Supplementary Figure 1. The trim-and-fill method was used to test our primary analysis of the publication bias

Supplementary Figure 2. Forest plot of the Subgroup analysis stratified by the modeling types

Supplementary Figure 3. Forest plot of the Subgroup analysis stratified by the marker types

Supplementary Figure 4. Forest plot of the Subgroup analysis stratified by the IVGC protocol types

Supplementary Figure 5. Forest plot of pooled AUCs of the optimal models selected from each study

Supplementary Figure 6. Sensitivity analysis

Supplementary Figure 7. Forest plot of pooled AUCs of prediction models of IVGC therapy response in TED

Supplementary Figure 8. The forest plot for the combined sensitivity

Supplementary Figure 9. The forest plot for the combined specificity

Supplementary Table 1. Procedure used to identify eligible studies

Supplementary Table 2. Characteristics of the studies included in the meta-analysis

## Declaration of interest

The authors declare that there is no conflict of interest that could be perceived as prejudicing the impartiality of the study reported.

## Data availability statement

Those with a legitimate need for data are welcome to contact the corresponding authors via email to request access.

## Author contribution statement

HFZ had full access to all the data in the study and takes responsibility for the integrity of the data and the accuracy of the data analysis; Concept and design: HFZ, HYZ; Acquisition, analysis, or interpretation of data:HYZ, SW, SYH; Drafting of the manuscript:HYZ, SW, SYH; Critical revision of the manuscript for important intellectual content:XFS, TNF, HFZ; *Statistical analysis:* TNF, HYZ; Supervision: HFZ, XQF, XFS.
