# Supplementary figures and images for: Prediction models of intravenous glucocorticoids therapy response in thyroid eye disease
Source: Eur Thyroid J. 2024 Aug 26;13(4):e240122. doi: 10.1530/ETJ-24-0122 (PMC11378126; doi:10.1530/ETJ-24-0122)

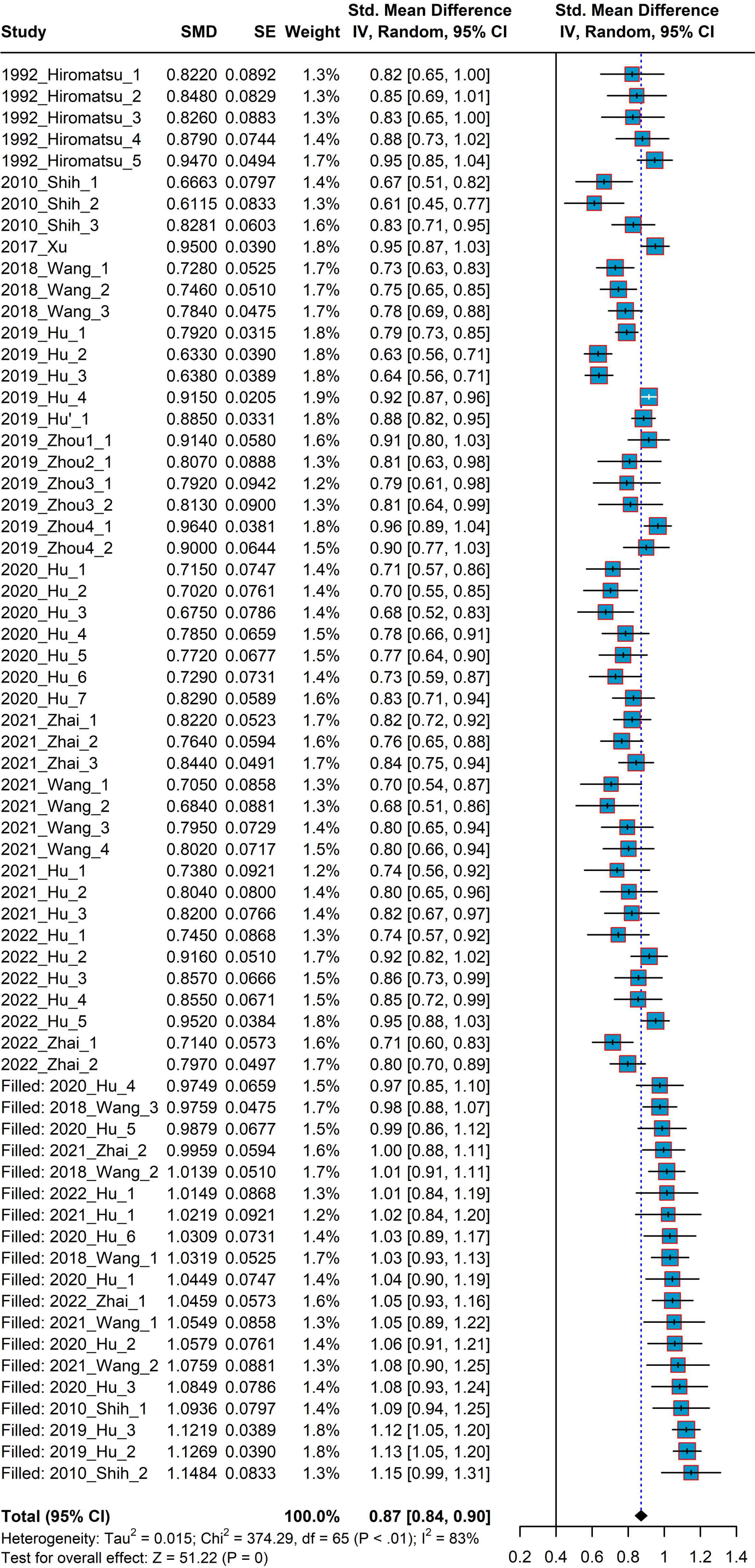

Supplement: Supplementary Figure 1. The trim-and-fill method was used to test our primary analysis of the publication bias [file supplementary_figure_1.pdf]

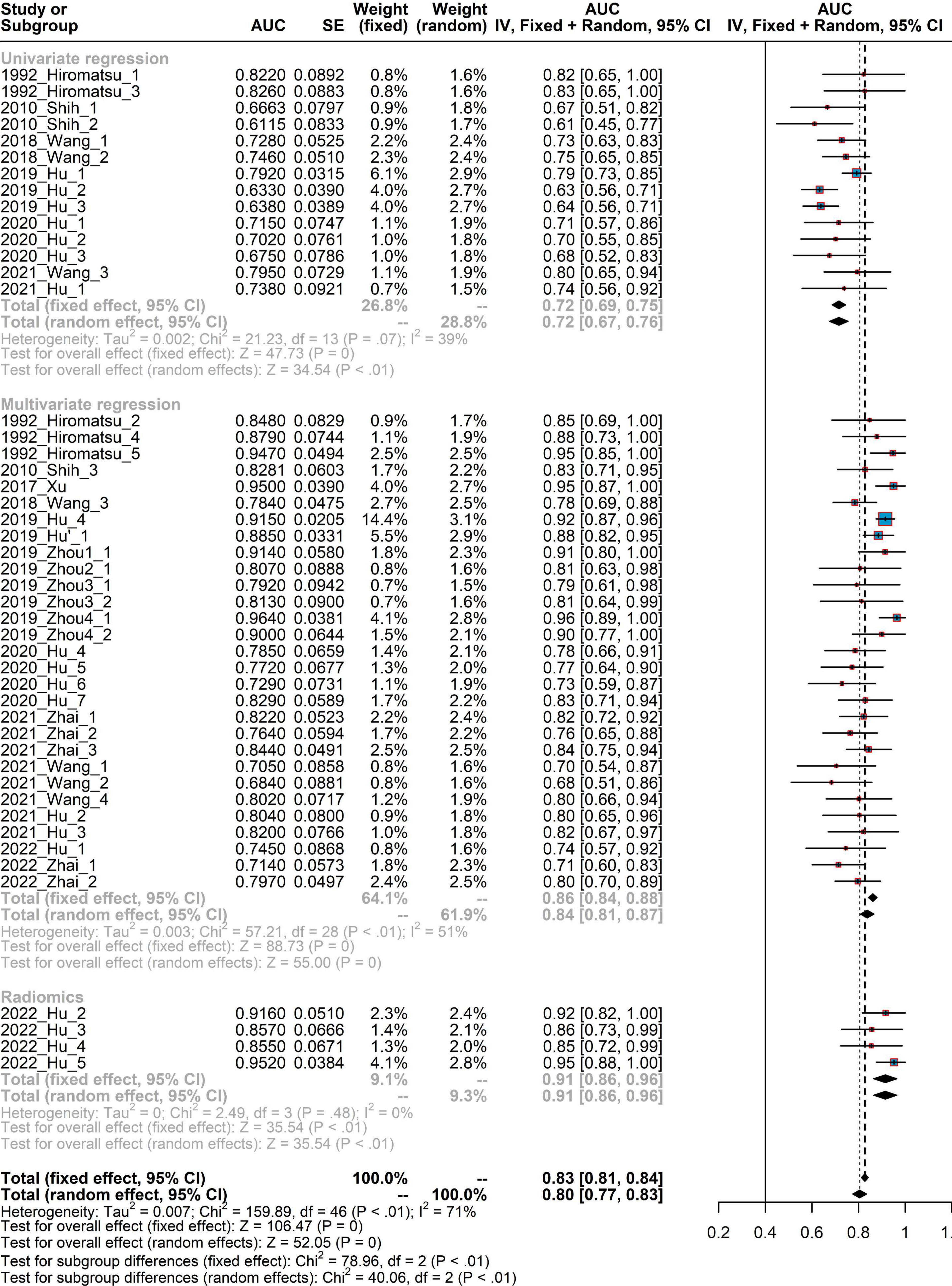

Supplement: Supplementary Figure 2. Forest plot of the Subgroup analysis stratified by the modeling types [file supplementary_figure_2.pdf]

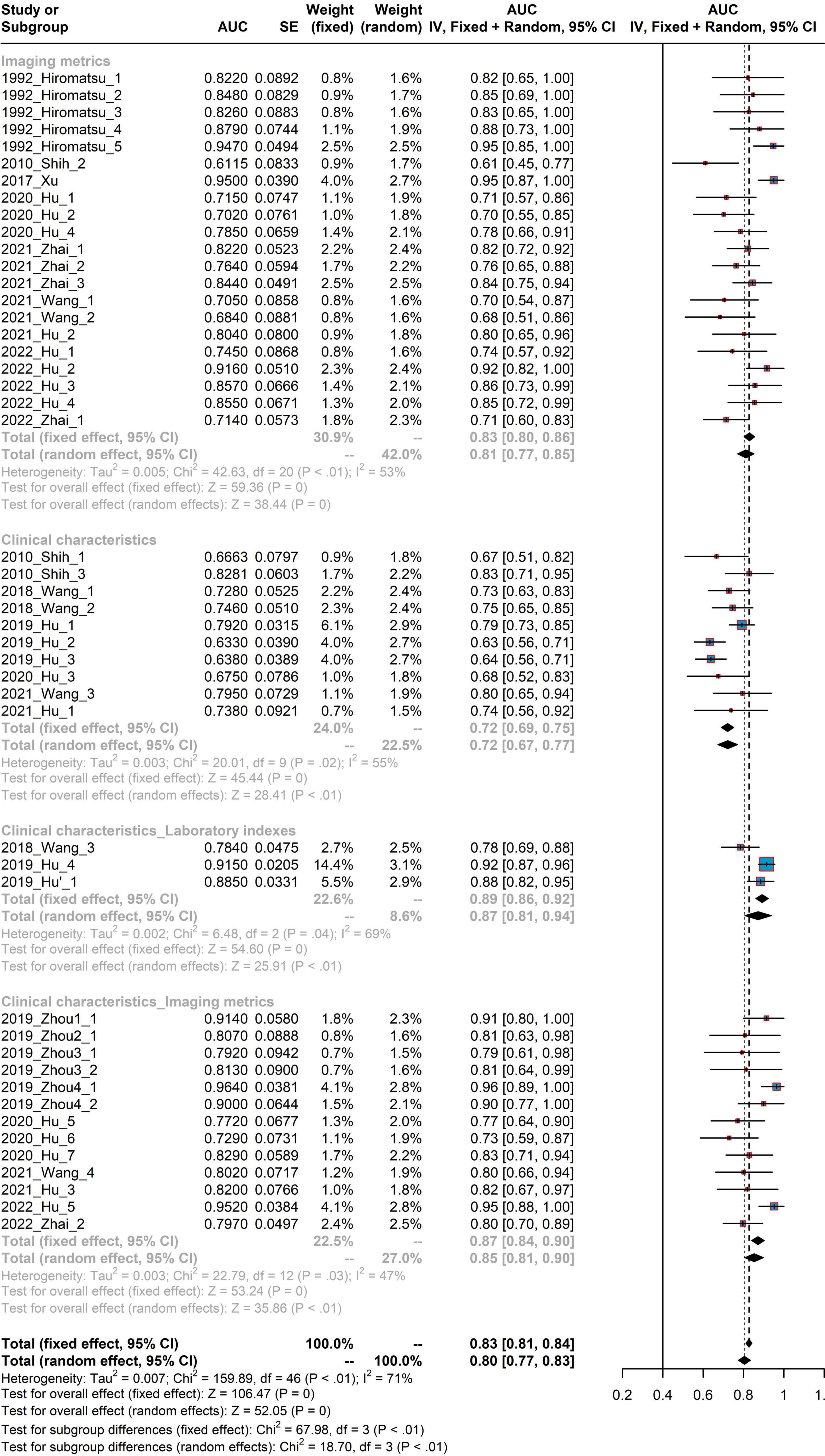

Supplement: Supplementary Figure 3. Forest plot of the Subgroup analysis stratified by the marker types [file supplementary_figure_3.pdf]

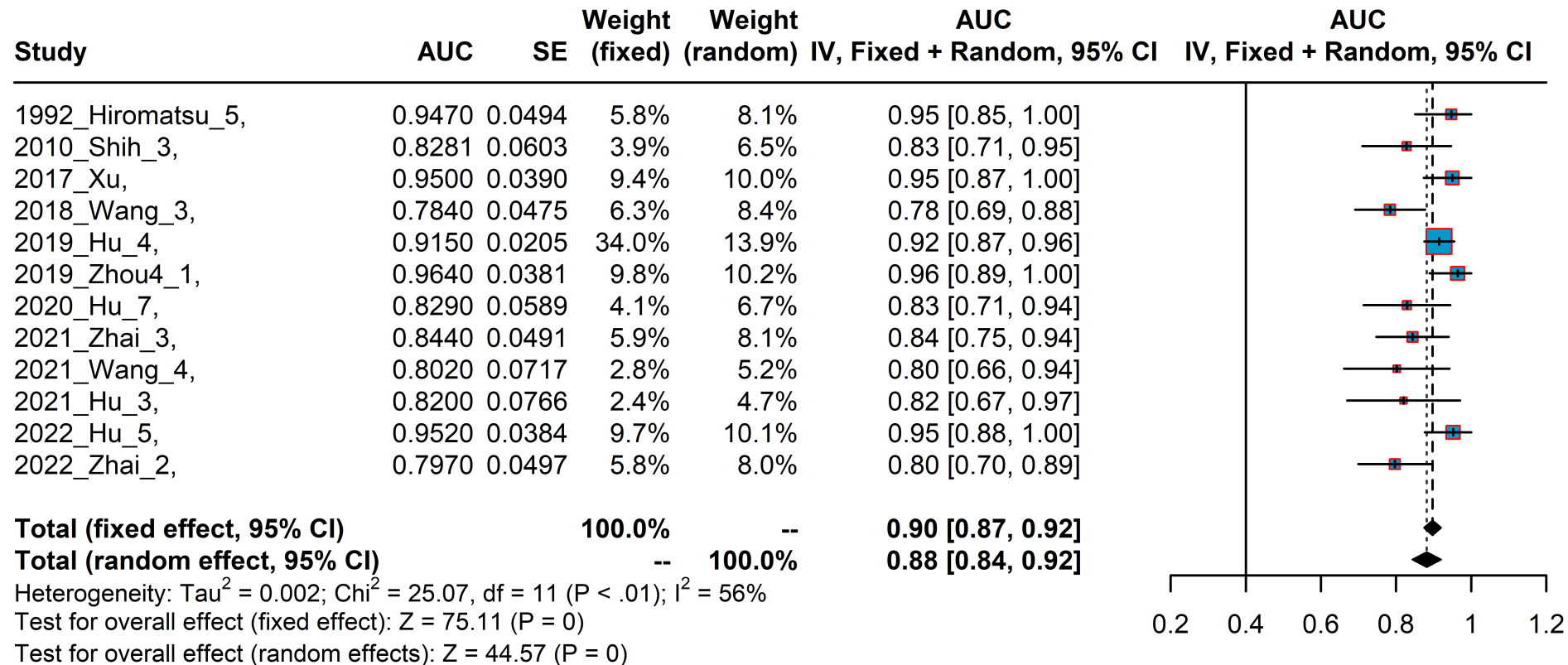

Supplement: Supplementary Figure 5. Forest plot of pooled AUCs of the optimal models selected from each study [file supplementary_figure_5.pdf]

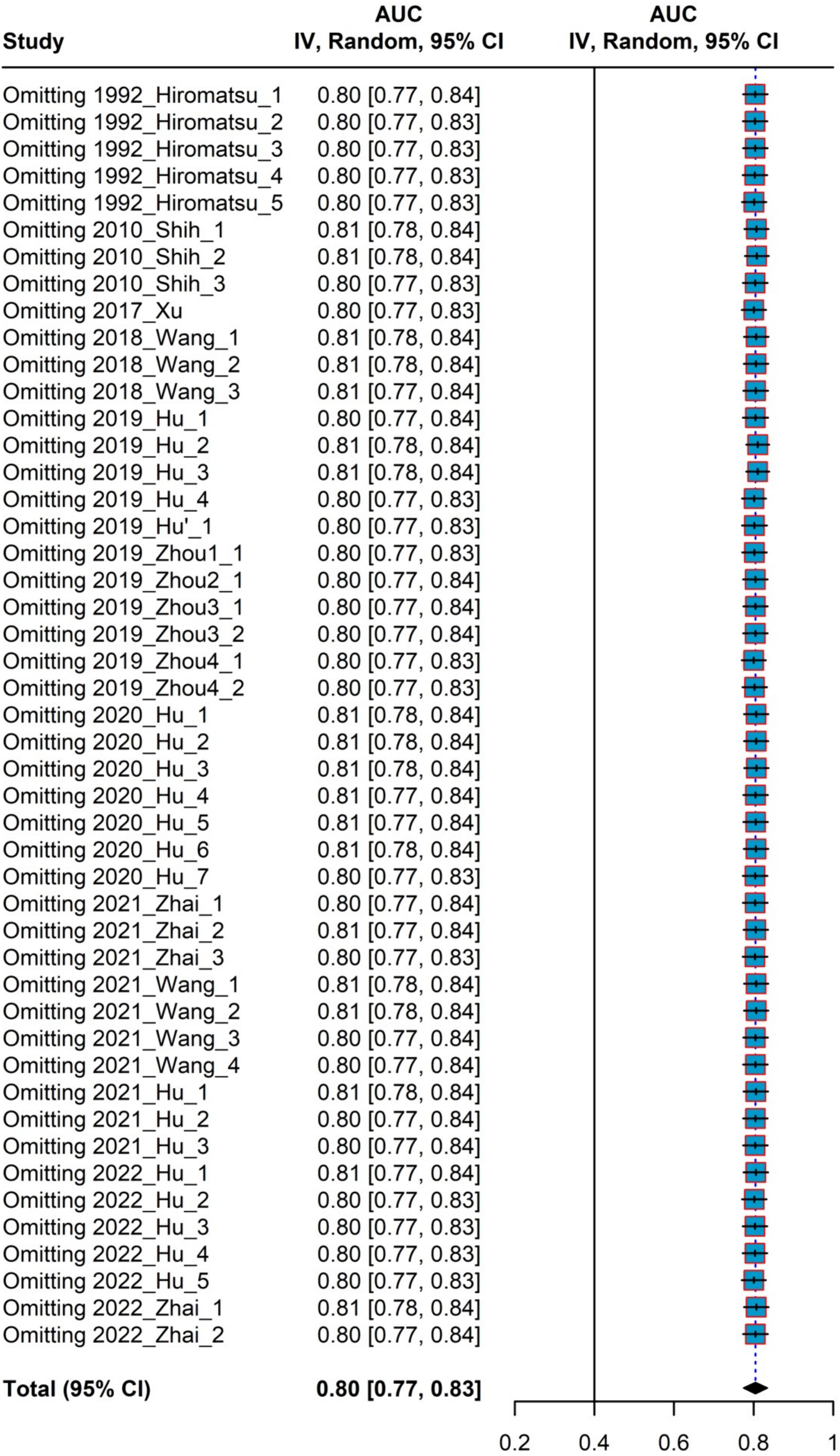

Supplement: Supplementary Figure 6. Sensitivity analysis [file supplementary_figure_6.pdf]

# Forest plot for true positive rate (sensitivity)

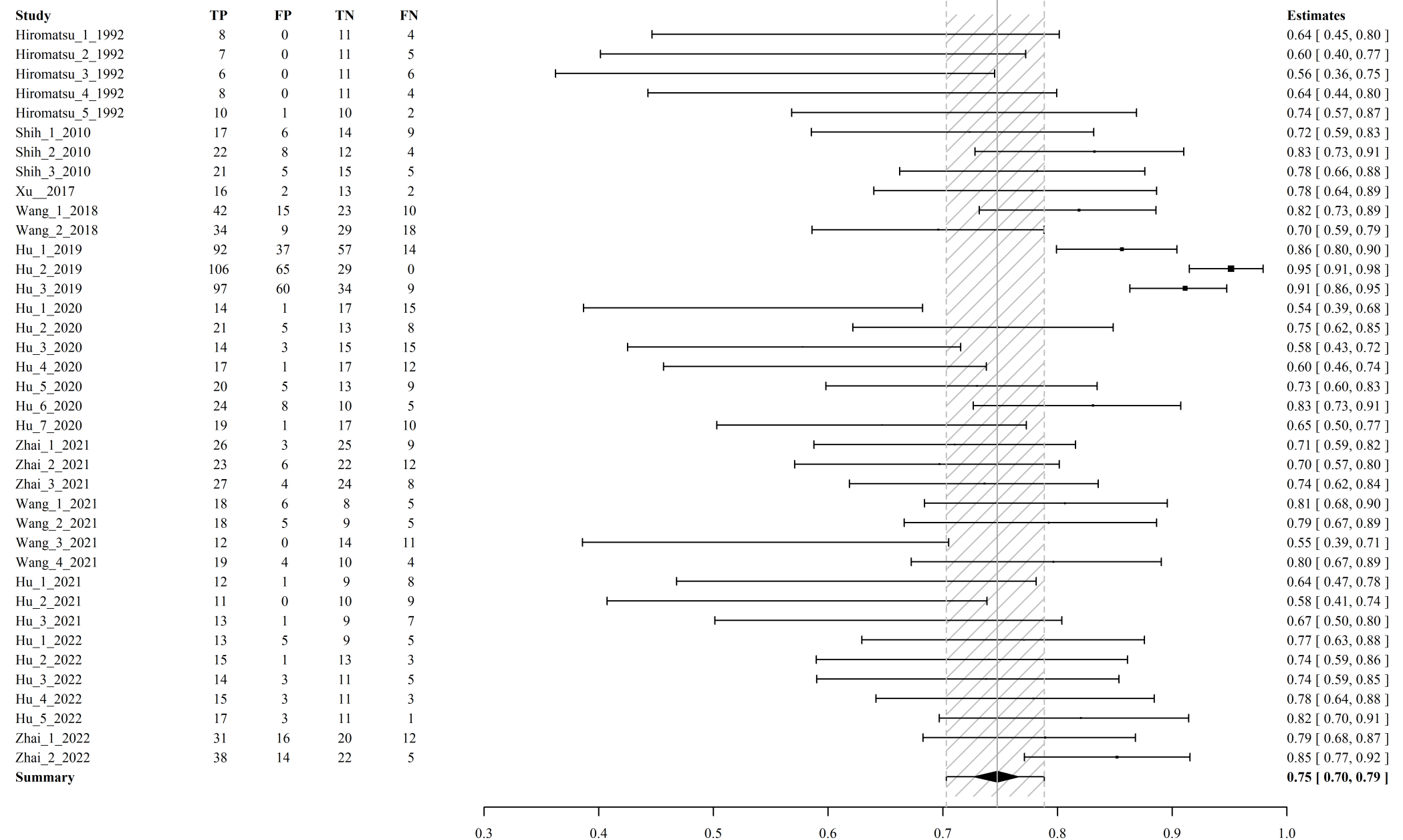

Supplement: Supplementary Figure 8. The forest plot for the combined sensitivity [file supplementary_figure_8.pdf]

# Forest plot for true negative rate (specificity)

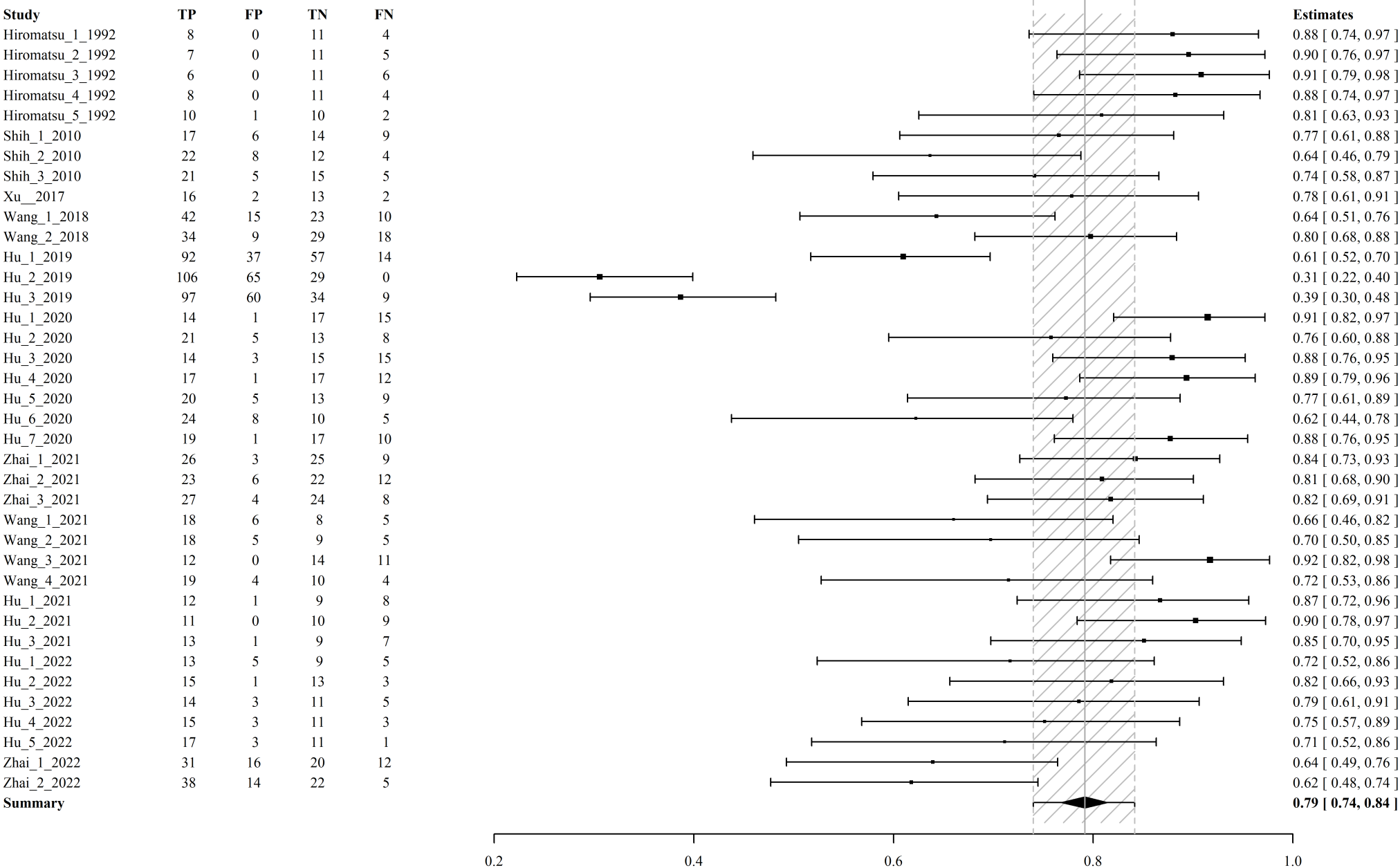

Supplement: Supplementary Figure 9. The forest plot for the combined specificity [file supplementary_figure_9.pdf]
