# Supplementary material for: Prediction models of intravenous glucocorticoids therapy response in thyroid eye disease
Source: Eur Thyroid J. 2024 Aug 26;13(4):e240122. doi: 10.1530/ETJ-24-0122 (PMC11378126; doi:10.1530/ETJ-24-0122)
Supplement: Supplementary Figure 4. Forest plot of the Subgroup analysis stratified by the IVGC protocol types [file supplementary_figure_4.pdf]

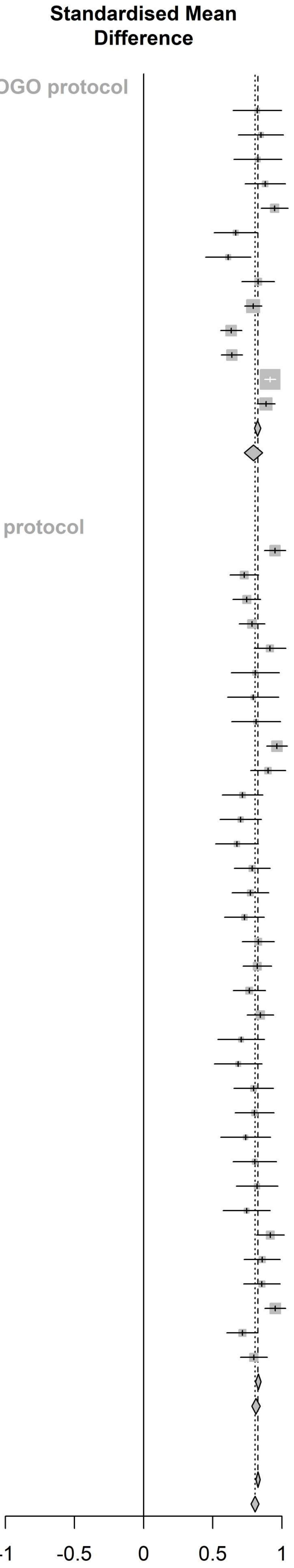

Study	SMD	SE(SMD)	SMD	95%-CI	Weight (common)	Weight (random)
**Group3_IVGC_protocol_type = non-EUGOGO protocol**						
1992_Hiromatsu_1	0.8220	0.0892	0.82	[0.65; 1.00]	0.8%	1.5%
1992_Hiromatsu_2	0.8480	0.0829	0.85	[0.69; 1.01]	0.9%	1.6%
1992_Hiromatsu_3	0.8260	0.0883	0.83	[0.65; 1.00]	0.8%	1.5%
1992_Hiromatsu_4	0.8790	0.0744	0.88	[0.73; 1.02]	1.1%	1.8%
1992_Hiromatsu_5	0.9470	0.0494	0.95	[0.85; 1.04]	2.5%	2.5%
2010_Shih_1	0.6663	0.0797	0.67	[0.51; 0.82]	0.9%	1.7%
2010_Shih_2	0.6115	0.0833	0.61	[0.45; 0.77]	0.9%	1.6%
2010_Shih_3	0.8281	0.0603	0.83	[0.71; 0.95]	1.7%	2.2%
2019_Hu_1	0.7920	0.0315	0.79	[0.73; 0.85]	6.1%	3.0%
2019_Hu_2	0.6330	0.0390	0.63	[0.56; 0.71]	4.0%	2.8%
2019_Hu_3	0.6380	0.0389	0.64	[0.56; 0.71]	4.0%	2.8%
2019_Hu_4	0.9150	0.0205	0.92	[0.87; 0.96]	14.4%	3.3%
2019_Hu'_1	0.8850	0.0331	0.88	[0.82; 0.95]	5.5%	3.0%
**Common effect model**			**0.82**	**[0.80; 0.85]**	**43.4%**	**.**
**Random effects model**			**0.79**	**[0.73; 0.86]**	**.**	**29.7%**
Heterogeneity: $I^2 = 86\%$, $\tau^2 = 0.0110$, $p < 0.01$						
**Group3_IVGC_protocol_type = EUGOGO protocol**						
2017_Xu	0.9500	0.0390	0.95	[0.87; 1.03]	4.0%	2.8%
2018_Wang_1	0.7280	0.0525	0.73	[0.63; 0.83]	2.2%	2.4%
2018_Wang_2	0.7460	0.0510	0.75	[0.65; 0.85]	2.3%	2.5%
2018_Wang_3	0.7840	0.0475	0.78	[0.69; 0.88]	2.7%	2.6%
2019_Zhou1_1	0.9140	0.0580	0.91	[0.80; 1.03]	1.8%	2.3%
2019_Zhou2_1	0.8070	0.0888	0.81	[0.63; 0.98]	0.8%	1.5%
2019_Zhou3_1	0.7920	0.0942	0.79	[0.61; 0.98]	0.7%	1.4%
2019_Zhou3_2	0.8130	0.0900	0.81	[0.64; 0.99]	0.7%	1.5%
2019_Zhou4_1	0.9640	0.0381	0.96	[0.89; 1.04]	4.1%	2.9%
2019_Zhou4_2	0.9000	0.0644	0.90	[0.77; 1.03]	1.5%	2.1%
2020_Hu_1	0.7150	0.0747	0.71	[0.57; 0.86]	1.1%	1.8%
2020_Hu_2	0.7020	0.0761	0.70	[0.55; 0.85]	1.0%	1.8%
2020_Hu_3	0.6750	0.0786	0.68	[0.52; 0.83]	1.0%	1.7%
2020_Hu_4	0.7850	0.0659	0.78	[0.66; 0.91]	1.4%	2.1%
2020_Hu_5	0.7720	0.0677	0.77	[0.64; 0.90]	1.3%	2.0%
2020_Hu_6	0.7290	0.0731	0.73	[0.59; 0.87]	1.1%	1.9%
2020_Hu_7	0.8290	0.0589	0.83	[0.71; 0.94]	1.7%	2.2%
2021_Zhai_1	0.8220	0.0523	0.82	[0.72; 0.92]	2.2%	2.4%
2021_Zhai_2	0.7640	0.0594	0.76	[0.65; 0.88]	1.7%	2.2%
2021_Zhai_3	0.8440	0.0491	0.84	[0.75; 0.94]	2.5%	2.5%
2021_Wang_1	0.7050	0.0858	0.70	[0.54; 0.87]	0.8%	1.6%
2021_Wang_2	0.6840	0.0881	0.68	[0.51; 0.86]	0.8%	1.5%
2021_Wang_3	0.7950	0.0729	0.80	[0.65; 0.94]	1.1%	1.9%
2021_Wang_4	0.8020	0.0717	0.80	[0.66; 0.94]	1.2%	1.9%
2021_Hu_1	0.7380	0.0921	0.74	[0.56; 0.92]	0.7%	1.5%
2021_Hu_2	0.8040	0.0800	0.80	[0.65; 0.96]	0.9%	1.7%
2021_Hu_3	0.8200	0.0766	0.82	[0.67; 0.97]	1.0%	1.8%
2022_Hu_1	0.7450	0.0868	0.74	[0.57; 0.92]	0.8%	1.6%
2022_Hu_2	0.9160	0.0510	0.92	[0.82; 1.02]	2.3%	2.5%
2022_Hu_3	0.8570	0.0666	0.86	[0.73; 0.99]	1.4%	2.0%
2022_Hu_4	0.8550	0.0671	0.85	[0.72; 0.99]	1.3%	2.0%
2022_Hu_5	0.9520	0.0384	0.95	[0.88; 1.03]	4.1%	2.9%
2022_Zhai_1	0.7140	0.0573	0.71	[0.60; 0.83]	1.8%	2.3%
2022_Zhai_2	0.7970	0.0497	0.80	[0.70; 0.89]	2.4%	2.5%
**Common effect model**			**0.83**	**[0.81; 0.85]**	**56.6%**	**.**
**Random effects model**			**0.81**	**[0.78; 0.84]**	**.**	**70.3%**
Heterogeneity: $I^2 = 54\%$, $\tau^2 = 0.0040$, $p < 0.01$						
**Common effect model**			**0.83**	**[0.81; 0.84]**	**100.0%**	**.**
**Random effects model**			**0.81**	**[0.78; 0.83]**	**.**	**100.0%**

Heterogeneity: $I^2 = 71\%$, $\tau^2 = 0.0059$, $p < 0.01$
Test for subgroup differences (common effect): $\chi^2_1 = 0.10$, df = 1 ($p = 0.76$)
Test for subgroup differences (random effects): $\chi^2_1 = 0.24$, df = 1 ($p = 0.63$)
