# Supplementary material for: Prediction models of intravenous glucocorticoids therapy response in thyroid eye disease
Source: Eur Thyroid J. 2024 Aug 26;13(4):e240122. doi: 10.1530/ETJ-24-0122 (PMC11378126; doi:10.1530/ETJ-24-0122)

Study	AUC	SE	Weight (common)	Weight (random)	AUC IV, Fixed + Random, 95% CI
1992_Hiromatsu_1	0.8220	0.0892	0.8%	1.5%	0.82 [0.65, 1.00]
1992_Hiromatsu_2	0.8480	0.0829	0.9%	1.6%	0.85 [0.69, 1.00]
1992_Hiromatsu_3	0.8260	0.0883	0.8%	1.5%	0.83 [0.65, 1.00]
1992_Hiromatsu_4	0.8790	0.0744	1.1%	1.8%	0.88 [0.73, 1.00]
1992_Hiromatsu_5	0.9470	0.0494	2.5%	2.5%	0.95 [0.85, 1.00]
2010_Shih_1	0.6663	0.0797	0.9%	1.7%	0.67 [0.51, 0.82]
2010_Shih_2	0.6115	0.0833	0.9%	1.6%	0.61 [0.45, 0.77]
2010_Shih_3	0.8281	0.0603	1.7%	2.2%	0.83 [0.71, 0.95]
2017_Xu	0.9500	0.0390	4.0%	2.8%	0.95 [0.87, 1.00]
2018_Wang_1	0.7280	0.0525	2.2%	2.4%	0.73 [0.63, 0.83]
2018_Wang_2	0.7460	0.0510	2.3%	2.5%	0.75 [0.65, 0.85]
2018_Wang_3	0.7840	0.0475	2.7%	2.6%	0.78 [0.69, 0.88]
2019_Hu_1	0.7920	0.0315	6.1%	3.0%	0.79 [0.73, 0.85]
2019_Hu_2	0.6330	0.0390	4.0%	2.8%	0.63 [0.56, 0.71]
2019_Hu_3	0.6380	0.0389	4.0%	2.8%	0.64 [0.56, 0.71]
2019_Hu_4	0.9150	0.0205	14.4%	3.3%	0.92 [0.87, 0.96]
2019_Hu'_1	0.8850	0.0331	5.5%	3.0%	0.88 [0.82, 0.95]
2019_Zhou1_1	0.9140	0.0580	1.8%	2.3%	0.91 [0.80, 1.00]
2019_Zhou2_1	0.8070	0.0888	0.8%	1.5%	0.81 [0.63, 0.98]
2019_Zhou3_1	0.7920	0.0942	0.7%	1.4%	0.79 [0.61, 0.98]
2019_Zhou3_2	0.8130	0.0900	0.7%	1.5%	0.81 [0.64, 0.99]
2019_Zhou4_1	0.9640	0.0381	4.1%	2.9%	0.96 [0.89, 1.00]
2019_Zhou4_2	0.9000	0.0644	1.5%	2.1%	0.90 [0.77, 1.00]
2020_Hu_1	0.7150	0.0747	1.1%	1.8%	0.71 [0.57, 0.86]
2020_Hu_2	0.7020	0.0761	1.0%	1.8%	0.70 [0.55, 0.85]
2020_Hu_3	0.6750	0.0786	1.0%	1.7%	0.68 [0.52, 0.83]
2020_Hu_4	0.7850	0.0659	1.4%	2.1%	0.78 [0.66, 0.91]
2020_Hu_5	0.7720	0.0677	1.3%	2.0%	0.77 [0.64, 0.90]
2020_Hu_6	0.7290	0.0731	1.1%	1.9%	0.73 [0.59, 0.87]
2020_Hu_7	0.8290	0.0589	1.7%	2.2%	0.83 [0.71, 0.94]
2021_Zhai_1	0.8220	0.0523	2.2%	2.4%	0.82 [0.72, 0.92]
2021_Zhai_2	0.7640	0.0594	1.7%	2.2%	0.76 [0.65, 0.88]
2021_Zhai_3	0.8440	0.0491	2.5%	2.5%	0.84 [0.75, 0.94]
2021_Wang_1	0.7050	0.0858	0.8%	1.6%	0.70 [0.54, 0.87]
2021_Wang_2	0.6840	0.0881	0.8%	1.5%	0.68 [0.51, 0.86]
2021_Wang_3	0.7950	0.0729	1.1%	1.9%	0.80 [0.65, 0.94]
2021_Wang_4	0.8020	0.0717	1.2%	1.9%	0.80 [0.66, 0.94]
2021_Hu_1	0.7380	0.0921	0.7%	1.5%	0.74 [0.56, 0.92]
2021_Hu_2	0.8040	0.0800	0.9%	1.7%	0.80 [0.65, 0.96]
2021_Hu_3	0.8200	0.0766	1.0%	1.8%	0.82 [0.67, 0.97]
2022_Hu_1	0.7450	0.0868	0.8%	1.6%	0.74 [0.57, 0.92]
2022_Hu_2	0.9160	0.0510	2.3%	2.5%	0.92 [0.82, 1.00]
2022_Hu_3	0.8570	0.0666	1.4%	2.0%	0.86 [0.73, 0.99]
2022_Hu_4	0.8550	0.0671	1.3%	2.0%	0.85 [0.72, 0.99]
2022_Hu_5	0.9520	0.0384	4.1%	2.9%	0.95 [0.88, 1.00]
2022_Zhai_1	0.7140	0.0573	1.8%	2.3%	0.71 [0.60, 0.83]
2022_Zhai_2	0.7970	0.0497	2.4%	2.5%	0.80 [0.70, 0.89]
**Total (fixed effect, 95% CI)**			**100.0%**	**--**	**0.83 [0.81, 0.84]**
**Total (random effect, 95% CI)**			**--**	**100.0%**	**0.81 [0.78, 0.83]**

Heterogeneity: $Tau^2 = 0.006$; $Chi^2 = 159.89$, df = 46 (P < .01); $I^2 = 71\%$
Test for overall effect (fixed effect): Z = 106.47 (P = 0)
Test for overall effect (random effects): Z = 55.52 (P = 0)

AUC
IV, Fixed + Random, 95% CI

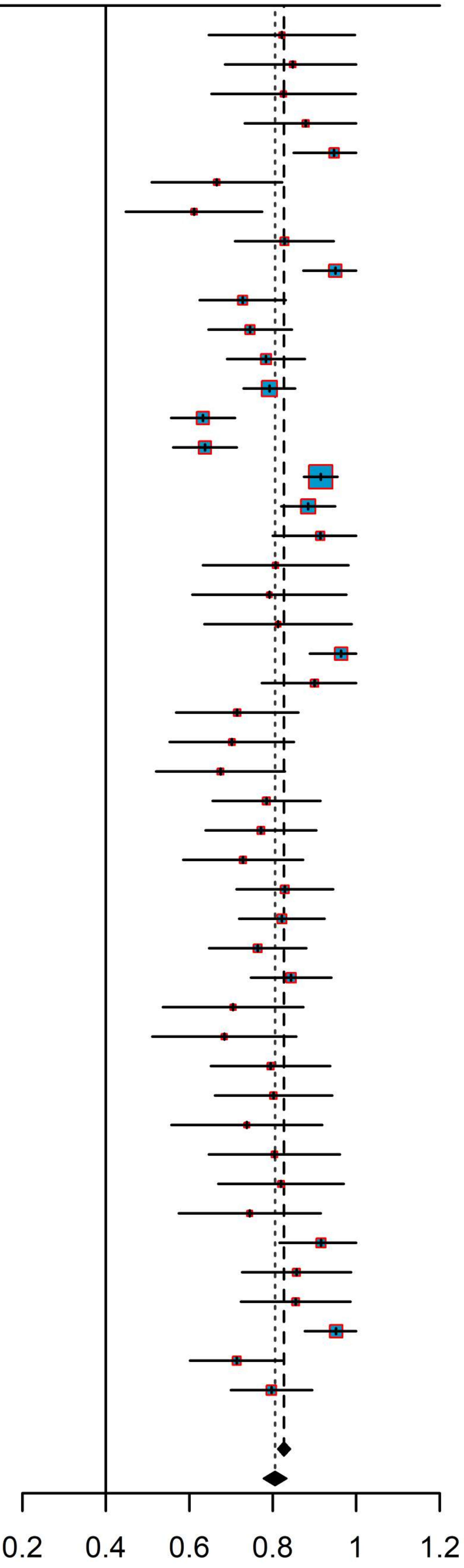

Supplement: Supplementary Figure 7. Forest plot of pooled AUCs of prediction models of IVGC therapy response in TED [file supplementary_figure_7.pdf]
