# Supplementary material for: Prediction models of intravenous glucocorticoids therapy response in thyroid eye disease
Source: Eur Thyroid J. 2024 Aug 26;13(4):e240122. doi: 10.1530/ETJ-24-0122 (PMC11378126; doi:10.1530/ETJ-24-0122)
Supplement: Supplementary Table 1. Procedure used to identify eligible studies [file supplementary_table_1.pdf]

Supplementary Table 1. Procedure used to identify eligible studies

**Database**	**Search terms**
Pubmed	(((((response prediction[Title/Abstract]) OR (response predict[Title/Abstract])) OR (prediction[Title/Abstract])) OR (predict[Title/Abstract])) OR (response[Title/Abstract])) AND ((((((("Glucocorticoids"[Mesh]) OR (Glucocorticoid[Title/Abstract])) OR (Glucocorticoid Effect[Title/Abstract])) OR (Effect, Glucocorticoid[Title/Abstract])) OR (Glucorticoid Effects[Title/Abstract])) OR (Effects, Glucorticoid[Title/Abstract])) AND ((((((((((((((((((((((((((((((((("Graves Ophthalmopathy"[Mesh]) OR (Ophthalmopathy, Graves[Title/Abstract])) OR (Ophthalmopathies, Thyroid-Associated[Title/Abstract])) OR (Thyroid-Associated Ophthalmopathies[Title/Abstract])) OR (Thyroid Associated Ophthalmopathies[Title/Abstract])) OR (Thyroid Eye Disease[Title/Abstract])) OR (Disease, Thyroid Eye[Title/Abstract])) OR (Eye Disease, Thyroid[Title/Abstract])) OR (Thyroid Eye Diseases[Title/Abstract])) OR (Thyroid-Associated Ophthalmopathy[Title/Abstract])) OR (Thyroid Associated Ophthalmopathy[Title/Abstract])) OR (Dysthyroid Ophthalmopathy[Title/Abstract])) OR (Dysthyroid Ophthalmopathies[Title/Abstract])) OR (Ophthalmopathy, Dysthyroid[Title/Abstract])) OR (Graves Eye Disease[Title/Abstract])) OR (Disease, Graves Eye[Title/Abstract])) OR (Eye Disease, Graves[Title/Abstract])) OR (Graves Orbitopathy[Title/Abstract])) OR (Orbitopathy, Graves[Title/Abstract])) OR (Ophthalmopathy, Thyroid-Associated[Title/Abstract])) OR (Ophthalmopathy, Thyroid Associated[Title/Abstract])) OR (Myopathic Ophthalmopathy[Title/Abstract])) OR (Myopathic Ophthalmopathies[Title/Abstract])) OR (Ophthalmopathy, Myopathic[Title/Abstract])) OR (Congestive Ophthalmopathy[Title/Abstract])) OR (Congestive Ophthalmopathies[Title/Abstract])) OR (Ophthalmopathy, Congestive[Title/Abstract])) OR (Edematous Ophthalmopathy[Title/Abstract])) OR (Edematous Ophthalmopathies[Title/Abstract])) OR (Ophthalmopathy, Edematous[Title/Abstract])) OR (Ophthalmopathy, Infiltrative[Title/Abstract])) OR (Infiltrative Ophthalmopathies[Title/Abstract])) OR (Infiltrative Ophthalmopathy[Title/Abstract])))
Web of science	TS=(Graves Ophthalmopathy OR Ophthalmopathy, Graves OR Ophthalmopathies, Thyroid-Associated OR Thyroid-Associated Ophthalmopathies OR Thyroid Associated Ophthalmopathies OR Thyroid Eye Disease OR Disease, Thyroid Eye OR Eye Disease, Thyroid OR Thyroid Eye Diseases OR Thyroid-Associated Ophthalmopathy OR Thyroid Associated Ophthalmopathy OR Dysthyroid Ophthalmopathy OR Dysthyroid Ophthalmopathies OR Ophthalmopathy, Dysthyroid OR Graves Eye Disease OR Disease, Graves Eye OR Eye Disease, Graves OR Graves Orbitopathy OR Orbitopathy, Graves OR Ophthalmopathy, Thyroid-Associated OR Ophthalmopathy, Thyroid Associated OR Myopathic Ophthalmopathy OR Myopathic Ophthalmopathies OR Ophthalmopathy, Myopathic OR Congestive Ophthalmopathy OR Congestive Ophthalmopathies OR Ophthalmopathy, Congestive OR Edematous Ophthalmopathy OR Edematous

	Ophthalmopathies OR Ophthalmopathy, Edematous OR Ophthalmopathy, Infiltrative OR Infiltrative Ophthalmopathies OR Infiltrative Ophthalmopathy) AND TS=(Glucocorticoids OR Glucocorticoid OR Glucocorticoid Effect OR Effect, Glucocorticoid OR Glucorticoid Effects OR Effects, Glucorticoid) AND TS=(Response Prediction OR Response Predict OR Prediction OR Rredict OR Response)
Embase	('graves ophthalmopathy':ab,ti OR 'ophthalmopathy, graves':ab,ti OR 'ophthalmopathies, thyroid-associated':ab,ti OR 'thyroid-associated ophthalmopathies':ab,ti OR 'thyroid associated ophthalmopathies':ab,ti OR 'thyroid eye disease':ab,ti OR 'disease, thyroid eye':ab,ti OR 'eye disease, thyroid':ab,ti OR 'thyroid eye diseases':ab,ti OR 'thyroid-associated ophthalmopathy':ab,ti OR 'thyroid associated ophthalmopathy':ab,ti OR 'dysthyroid ophthalmopathy':ab,ti OR 'dysthyroid ophthalmopathies':ab,ti OR 'ophthalmopathy, dysthyroid':ab,ti OR 'graves eye disease':ab,ti OR 'disease, graves eye':ab,ti OR 'eye disease, graves':ab,ti OR 'graves orbitopathy':ab,ti OR 'orbitopathy, graves':ab,ti OR 'ophthalmopathy, thyroid-associated':ab,ti OR 'ophthalmopathy, thyroid associated':ab,ti OR 'myopathic ophthalmopathy':ab,ti OR 'myopathic ophthalmopathies':ab,ti OR 'ophthalmopathy, myopathic':ab,ti OR 'congestive ophthalmopathy':ab,ti OR 'congestive ophthalmopathies':ab,ti OR 'ophthalmopathy, congestive':ab,ti OR 'edematous ophthalmopathy':ab,ti OR 'edematous ophthalmopathies':ab,ti OR 'ophthalmopathy, edematous':ab,ti OR 'ophthalmopathy, infiltrative':ab,ti OR 'infiltrative ophthalmopathies':ab,ti OR 'infiltrative ophthalmopathy':ab,ti) AND (glucocorticoids:ab,ti OR glucocorticoid:ab,ti OR 'glucocorticoid?effect':ab,ti OR 'effect,?glucocorticoid':ab,ti OR 'glucorticoid effects':ab,ti OR 'effects, glucorticoid':ab,ti) AND ('response prediction':ab,ti OR 'response predict':ab,ti OR prediction:ab,ti OR predict:ab,ti OR response:ab,ti)
