# Supplementary material for: Prediction models of intravenous glucocorticoids therapy response in thyroid eye disease
Source: Eur Thyroid J. 2024 Aug 26;13(4):e240122. doi: 10.1530/ETJ-24-0122 (PMC11378126; doi:10.1530/ETJ-24-0122)
Supplement: Supplementary Table 2. Characteristics of the studies included in the meta-analysis [file supplementary_table_2.pdf]

Supplementary Table 2. Characteristics of the studies included in the meta-analysis

Study No.	Year	Author	Region	Model No.	Number of participants			Model content	Modeling type	Marker type	IVGC protocol type	AUC	Sensitivity	Specificity
					Total	Responsive	Unresponsive							
1	1992	Hiromatsu	Fukuoka, Japan	1992_Hiromatsu_1	23	12	11	Thickness of right medial rectus	univariate regression	Imaging metrics	non-EUGOGO protocol	0.822	0.667	1
				1992_Hiromatsu_2	23	12	11	SI-EOM	multivariate regression	Imaging metrics	non-EUGOGO protocol	0.848	0.583	1
				1992_Hiromatsu_3	23	12	11	SI-OCT	univariate regression	Imaging metrics	non-EUGOGO protocol	0.826	0.5	1
				1992_Hiromatsu_4	23	12	11	SI-EOM + SI-OCT	multivariate regression	Imaging metrics	non-EUGOGO protocol	0.879	0.667	1
				1992_Hiromatsu_5	23	12	11	Thickness of right medial rectus + SI-EOM	multivariate regression	Imaging metrics	non-EUGOGO protocol	0.947	0.833	0.909
2	2010	Shih	Taiwan, China	2010_Shih_1	46	26*	20*	CAS ≥ 3	univariate regression	Clinical characteristics	non-EUGOGO protocol	0.6663	0.6471	0.7209
				2010_Shih_2	46	26*	20*	mean temperature ≥ 32.87°	univariate regression	Imaging metrics	non-EUGOGO protocol	0.6115	0.85	0.6087

				2010_Shih_3	46	26*	20*	Prognostic score=CAS + 2.6 mean temperature-2.3 sex ≥ 87	multivariate regression	Clinical characteristics	non-EUGOGO protocol	0.8281	0.8077	0.7647
3	2017	Xu	Guangdong, China	2017_Xu	33	18	15	Exophthalmos ≥ 20.78 + inferior rectus/fat ratio ≥ 1.42	multivariate regression	Imaging metrics	EUGOGO protocol	0.95	0.867	0.889
4	2018	Wang	Shanghai, China	2018_Wang_1	90	52	38	CAS ＞ 2.5	univariate regression	Clinical characteristics	EUGOGO protocol	0.728	0.805	0.615
				2018_Wang_2	90	52	38	disease duration ≤ 13 months	univariate regression	Clinical characteristics	EUGOGO protocol	0.746	0.658	0.769
				2018_Wang_3	90	52	38	disease duration + restoration of euthyroidism	multivariate regression	Clinical characteristics + Laboratory indexes	EUGOGO protocol	0.784	/	/
5	2019	Hu	Xi'an, China	2019_Hu_1	200	106	94	CAS ≥ 2.5	univariate regression	Clinical characteristics	non-EUGOGO protocol	0.792	0.871	0.602
				2019_Hu_2	200	106	94	Disease duration ≥ 15 months	univariate regression	Clinical characteristics	non-EUGOGO protocol	0.633	1	0.306
				2019_Hu_3	200	106	94	pretreatment triglycerides > 158.9 mg/dL	univariate regression	Clinical characteristics	non-EUGOGO protocol	0.638	0.919	0.359
				2019_Hu_4	200	106	94	CAS + disease duration + positive pretreatment	multivariate regression	Clinical characteristics + Laboratory indexes	non-EUGOGO protocol	0.915	/	/

								TRAB(> 9.31 U/L) + high pretreatment triglyceride(≥ 150 mg/dL) + elevated TSH during treatment(> 5 μIU/mL)						
				2019_Hu'_1	102	57	44	CAS + disease duration + positive pretreatment TRAB(> 9.31 U/L) + high pretreatment triglyceride(≥ 150 mg/dL) + elevated TSH during treatment(>5 μIU/mL)	multivariate regression	Clinical characteristics + Laboratory indexes	non-EUGOGO protocol	0.885	/	/
6	2019	Zhou	Shanghai, China	2019_Zhou1_1	26	21	5	CAS + disease duration + SIR of most inflamed muscle ≥ 0.78	multivariate regression	Clinical characteristics + Imaging metrics	EUGOGO protocol	0.914	/	/
				2019_Zhou2_1	24	15	9	CAS + disease duration + SIR of medial	multivariate regression	Clinical characteristics + Imaging metrics	EUGOGO protocol	0.807	/	/

								rectus ≥ 0.54						
				2019_Zhou3_1	24	12	12	CAS + disease duration + SIR of most inflamed muscle ≥ 0.5	multivariate regression	Clinical characteristics + Imaging metrics	EUGOGO protocol	0.792	/	/
				2019_Zhou3_2	24	12	12	CAS + disease duration + SIR of medial rectus ≥ 0.41	multivariate regression	Clinical characteristics + Imaging metrics	EUGOGO protocol	0.813	/	/
				2019_Zhou4_1	24	14	10	CAS + disease duration + SIR of medial rectus ≥ 0.54	multivariate regression	Clinical characteristics + Imaging metrics	EUGOGO protocol	0.964	/	/
				2019_Zhou4_2	24	14	10	CAS + disease duration + SIR of most inflamed muscle ≥ 0.35	multivariate regression	Clinical characteristics + Imaging metrics	EUGOGO protocol	0.9	/	/
7	2020	Hu	Nanjing, China	2020_Hu_1	47	29	18	$EOM\text{-}SIR_{min}$ ≥ 1.43	univariate regression	Imaging metrics	EUGOGO protocol	0.715	0.466	0.917
				2020_Hu_2	47	29	18	LGH/OFT ≥ 1.65	univariate regression	Imaging metrics	EUGOGO protocol	0.702	0.707	0.722
				2020_Hu_3	47	29	18	disease duration ≤ 3.5	univariate regression	Clinical characteristics	EUGOGO protocol	0.675	0.483	0.833
				2020_Hu_4	47	29	18	$EOM\text{-}SIR_{min}$ ≥ 1.43 + LGH/OFT ≥	multivariate regression	Imaging metrics	EUGOGO protocol	0.785	0.569	0.944

								1.65						
				2020_Hu_5	47	29	18	EOM-SIR_{min} ≥ 1.43 + disease duration ≤ 3.5	multivariate regression	Clinical characteristics + Imaging metrics	EUGOGO protocol	0.772	0.69	0.75
				2020_Hu_6	47	29	18	LGH/OFT ≥ 1.65+ disease duration ≤ 3.5	multivariate regression	Clinical characteristics + Imaging metrics	EUGOGO protocol	0.729	0.828	0.528
				2020_Hu_7	47	29	18	EOM-SIR_{min}≥ 1.43 + LGH/OFT ≥ 1.65+ DD ≤ 3.5	multivariate regression	Clinical characteristics + Imaging metrics	EUGOGO protocol	0.829	0.638	0.917
8	2021	Zhai	Hubei, China	2021_Zhai_1	63	35	28	EOM-WF_{max} ≥ 91.25	multivariate regression	Imaging metrics	EUGOGO protocol	0.822	0.729	0.893
				2021_Zhai_2	63	35	28	EOM-$T2RT_{mean}$ ≥ 77.1	multivariate regression	Imaging metrics	EUGOGO protocol	0.764	0.671	0.768
				2021_Zhai_3	63	35	28	EOM-$T2RT_{mean}$ ≥ 77.1 + EOM-WF_{max} ≥ 91.25	multivariate regression	Imaging metrics	EUGOGO protocol	0.844	0.771	0.875
9	2021	Wang	Nanjing, China	2021_Wang_1	37	23	14	EOM–Entropy ≤ 3.855	multivariate regression	Imaging metrics	EUGOGO protocol	0.705	0.804	0.607
				2021_Wang_2	37	23	14	EOM–Uniformity ≥ 0.081	multivariate regression	Imaging metrics	EUGOGO protocol	0.684	0.804	0.643
				2021_Wang_3	37	23	14	disease duration ≤ 3.0	univariate regression	Clinical characteristics	EUGOGO protocol	0.795	0.522	1
				2021_Wang_4	37	23	14	EOM–Entropy ≤	multivariate regression	Clinical characteristics	EUGOGO protocol	0.802	0.826	0.679

								3.855 + EOM–Uniformity ≥ 0.081 + disease duration ≤ 3.0		+ Imaging metrics				
10	2021	Hu	Nanjing, China	2021_Hu_1	30	20	10	disease duration ≤ 4.5	univariate regression	Clinical characteristics	EUGOGO protocol	0.738	0.6	0.9
				2021_Hu_2	30	20	10	EOM-$T2RT_{min}$ ≥ 54.3	multivariate regression	Imaging metrics	EUGOGO protocol	0.804	0.575	1
				2021_Hu_3	30	20	10	EOM-$T2RT_{min}$ ≥ 54.3+ disease duration ≤ 4.5	multivariate regression	Clinical characteristics + Imaging metrics	EUGOGO protocol	0.82	0.65	0.95
11	2022	Hu	Nanjing, China	2022_Hu_1	32	18	14	EOM-SIR_{min}	multivariate regression	Imaging metrics	EUGOGO protocol	0.745	0.0075	0.00643
				2022_Hu_2	32	18	14	EOM-T2WI-radiomics model (logistic regression)	radiomics	Imaging metrics	EUGOGO protocol	0.916	0.00861	0.00893
				2022_Hu_3	32	18	14	EOM-T2WI-radiomics model (decision tree)	radiomics	Imaging metrics	EUGOGO protocol	0.857	0.75	0.821
				2022_Hu_4	32	18	14	EOM-T2WI-radiomics model（support vector machine）	radiomics	Imaging metrics	EUGOGO protocol	0.855	0.861	0.821

				2022_Hu_5	32	18	14	Nomogram: EOM-T2WI-radiomics model (logistic regression) + disease duration	radiomics	Clinical characteristics + Imaging metrics	EUGOGO protocol	0.952	0.917	0.821
12	2022	Zhai	Wuhan, China	2022_Zhai_1	79	43	36	FS-95th + FS-kurtosis	multivariate regression	Imaging metrics	EUGOGO protocol	0.714	0.721	0.556
				2022_Zhai_2	79	43	36	Disease duration + FS-95th + FS-kurtosis	multivariate regression	Clinical characteristics + Imaging metrics	EUGOGO protocol	0.797	0.884	0.625

Abbreviations: T2RT：T2 relaxation time; WF：water fraction; SI：signal intensity; SIR：signal intensity ratio; T2WI：T2-weighted imaging; OCT: orbital connective tissue; EOM: extraocular muscle; LGH: lacrimal gland herniation; OFT: orbital fat thickness; DD: disease duration; AUC: the area under curve; EUGOGO: European Group on Graves' Orbitopathy (total 4.5g methylprednisolone: 500 mg weekly for the first 6 weeks, followed by 250 mg weekly for the next 6 weeks); IVGC: intravenous glucocorticoid.

***:** The estimated number of unreported cases was calculated from the proportion of individuals who responded compared to those who did not, among the total population.

References

1. Hiromatsu Y, Kojima K, Ishisaka N, et al. Role of Magnetic Resonance Imaging in Thyroid Associated Ophthalmopathy: Its Predictive Value for Therapeutic Outcome of Immunosuppressive Therapy. Thyroid. 1992;2(4):299-305. (https://doi.org/10.1089/thy.1992.2.299)
2. Shih SR, Li HY, Hsiao YL, Chang TC. The application of temperature measurement of the eyes by digital infrared thermal imaging as a prognostic factor of

methylprednisolone pulse therapy for Graves' ophthalmopathy. Acta Ophthalmologica. 2010;88(5):e154-e159. (https://doi.org/10.1111/j.1755-3768.2010.01941.x)

3. Xu L, Li L, Xie C, Guan M, Xue Y. Thickness of Extraocular Muscle and Orbital Fat in MRI Predicts Response to Glucocorticoid Therapy in Graves' Ophthalmopathy. International Journal of Endocrinology. 2017;2017:1-8. (https://doi.org/10.1155/2017/3196059)

4. Wang Y, Zhang S, Zhang Y, et al. A single-center retrospective study of factors related to the effects of intravenous glucocorticoid therapy in moderate-to-severe and active thyroid-associated ophthalmopathy. BMC Endocr Disord. 2018;18(1):13. (https://doi.org/10.1186/s12902-018-0240-8)

5. Hu S, Wang Y, He M, Zhang M, Ding X, Shi B. Factors associated with the efficacy of intravenous methylprednisolone in moderate-to-severe and active thyroid-associated ophthalmopathy: A singlecentre retrospective study: XXXX. Clin Endocrinol. 2019;90(1):175-183. (https://doi.org/10.1111/cen.13855)

6. Zhou M, Shen L, Jiao Q, et al. Role of Magnetic Resonance Imaging in the Assessment of Active Thyroid-Associated Ophthalmopathy Patients with Long Disease Duration. Endocrine Practice. 2019;25(12):1268-1278. (https://doi.org/10.4158/EP-2019-0133)

7. Hu H, Xu XQ, Chen L, et al. Predicting the response to glucocorticoid therapy in thyroid-associated ophthalmopathy: mobilizing structural MRI-based quantitative measurements of orbital tissues. Endocrine. 2020;70(2):372-379. (https://doi.org/10.1007/s12020-020-02367-5)

8. Zhai L, Luo B, Wu H, et al. Prediction of treatment response to intravenous glucocorticoid in patients with thyroid-associated ophthalmopathy using T2

mapping and T2 IDEAL. European Journal of Radiology. 2021;142:109839. (https://doi.org/10.1016/j.ejrad.2021.109839)

9. Wang YY, Wu Q, Chen L, et al. Texture analysis of orbital magnetic resonance imaging for monitoring and predicting treatment response to glucocorticoids in patients with thyroid-associated ophthalmopathy. Endocrine Connections. 2021;10(7):676-684. (https://doi.org/10.1530/EC-21-0162)

10. Hu H, Chen HH, Chen W, et al. T2 mapping histogram at extraocular muscles for predicting the response to glucocorticoid therapy in patients with thyroid-associated ophthalmopathy. Clinical Radiology. 2021;76(2):159.e1-159.e8. (https://doi.org/10.1016/j.crad.2020.09.005)

11. Hu H, Chen L, Zhang J, et al. T2-Weighted MR Imaging-Derived Radiomics for Pretreatment Determination of Therapeutic Response to Glucocorticoid in Patients With Thyroid-Associated Ophthalmopathy: Comparison With Semiquantitative Evaluation. Magnetic Resonance Imaging. 2022;56(3):862-872. (https://doi.org/10.1002/jmri.28088)

12. Zhai L, Wang Q, Liu P, Luo B, Yuan G, Zhang J. T2 Mapping with and without Fat-Suppression to Predict Treatment Response to Intravenous Glucocorticoid Therapy for Thyroid-Associated Ophthalmopathy. Korean J Radiol. 2022;23(6):664-673. (https://doi.org/10.3348/kjr.2021.0627)
